# A globally relevant change taxonomy and evidence‐based change framework for land monitoring

**DOI:** 10.1111/gcb.16346

**Published:** 2022-09-01

**Authors:** Richard M. Lucas, Sophia German, Graciela Metternicht, Rebecca K. Schmidt, Christopher J. Owers, Suzanne M. Prober, Anna E. Richards, Sally Tetreault‐Campbell, Kristen J. Williams, Norman Mueller, Belle Tissott, Sean M. T. Chua, Alison Cowood, Terry Hills, Dayani Gunawardana, Alexis McIntyre, Sebastien Chognard, Clive Hurford, Carole Planque, Suvarna Punalekar, Daniel Clewley, Ruth Sonnenschein, Nicholas J. Murray, Ioannis Manakos, Palma Blonda, Kate Owers, Stephen Roxburgh, Heather Kay, Peter Bunting, Claire Horton

**Affiliations:** ^1^ Department of Geography and Earth Sciences Aberystwyth University Aberystwyth Ceredigion UK; ^2^ Earth and Sustainability Science Research Centre University of New South Wales Sydney New South Wales Australia; ^3^ CSIRO Land and Water Canberra Australian Capital Territory Australia; ^4^ CSIRO Land and Water Wembley Western Australia Australia; ^5^ CSIRO Land and Water Winnellie Northern Territory Australia; ^6^ CSIRO Land and Water Clayton South Victoria Australia; ^7^ Geoscience Australia Symonston Australian Capital Territory Australia; ^8^ Department of Climate Change, Energy, the Environment and Water Canberra Australian Capital Territory Australia; ^9^ Centre for Geospatial Applications Plymouth Marine Laboratory Plymouth Devon UK; ^10^ Institute for Earth Observation Eurac Research Bozen/Bolzano Italy; ^11^ College of Science and Engineering James Cook University Townsville Queensland Australia; ^12^ Centre for Research and Technology Hellas (CERTH) Thermi Greece; ^13^ Istituto sull'Inquinamento Atmosferico, CNR‐IIA, presso Dipartiment, Interateneo di Fisica Università of Bari Bari Italy; ^14^ Economy, Skills and Natural Resourcs (ESNR), Welsh Government Wales Ceredigion UK

**Keywords:** change, climate, Earth observations, economy, impacts, land cover, policy, pressures

## Abstract

A globally relevant and standardized taxonomy and framework for consistently describing land cover change based on evidence is presented, which makes use of structured land cover taxonomies and is underpinned by the Driver‐Pressure‐State‐Impact‐Response (DPSIR) framework. The Global Change Taxonomy currently lists 246 classes based on the notation ‘impact (pressure)’, with this encompassing the consequence of observed change and associated reason(s), and uses scale‐independent terms that factor in time. Evidence for different impacts is gathered through temporal comparison (e.g., days, decades apart) of land cover classes constructed and described from Environmental Descriptors (EDs; state indicators) with pre‐defined measurement units (e.g., m, %) or categories (e.g., species type). Evidence for pressures, whether abiotic, biotic or human‐influenced, is similarly accumulated, but EDs often differ from those used to determine impacts. Each impact and pressure term is defined separately, allowing flexible combination into ‘impact (pressure)’ categories, and all are listed in an openly accessible glossary to ensure consistent use and common understanding. The taxonomy and framework are globally relevant and can reference EDs quantified on the ground, retrieved/classified remotely (from ground‐based, airborne or spaceborne sensors) or predicted through modelling. By providing capacity to more consistently describe change processes—including land degradation, desertification and ecosystem restoration—the overall framework addresses a wide and diverse range of local to international needs including those relevant to policy, socioeconomics and land management. Actions in response to impacts and pressures and monitoring towards targets are also supported to assist future planning, including impact mitigation actions.

## Graphical abstract

The globally relevant and standardized change taxonomy and framework provides consistent descriptions of land cover dynamics, is underpinned by the Driver‐Pressure‐State‐Impact‐Response (DPSIR) framework and utilizes structured land cover taxonomies. The taxonomy defines and describes 246 change categories that use the notation ‘impacts (pressures)’. The taxonomy and evidence‐based change framework are scalable in space and time and can be applied to describe past and future (predicted or planned) changes, including at the ground, from Earth observations and through predictive models.

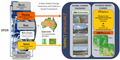

## 1 INTRODUCTION

Since the Agricultural and Industrial Revolutions, and particularly from the mid‐20th century onwards, humankind has directly or indirectly transformed the entire Earth's surface (Zalasiewicz et al., 2015) and its climate (Findell et al., 2017) and compromised the capacity of natural ecosystems to maintain biodiversity and function (Nicholson et al., 2021). The expansion of agriculture, forestry and human habitation, in particular, has resulted in major conversions or modifications of land cover and has been a primary contributor to the loss and degradation of ecosystems globally. In recent decades, awareness and understanding of land cover changes and their impacts have increased, partly due to Earth observing satellites with global monitoring capacity. However, more emphasis has been placed on documenting change rather than exploring connections and interactions with the different drivers and pressures (Winkler et al., 2021). This understanding is now critical if we are to reverse the damage inflicted on the Earth's system, particularly as change is driven largely by socioeconomic factors and climate, with both directing or being directed by policy and land management decisions. Given the diversity and complexity of the drivers and resulting pressures that lead to land cover conversions or modifications, a consistent and understandable framework for describing and quantifying change is essential. Such a framework is currently lacking, and this is hindering our ability to address major issues and challenges facing both humans and nature.

Land cover describes the physical and biological cover of the Earth's surface and differs from land use, which details the economic and social functions of land to meet demands for food, fibre, shelter and natural resources (Diogo & Koomen, 2016). Comparison of land covers over time provides the basis for describing and quantifying change. Change is an important descriptor from local to global levels as it informs (i) how current landscapes have evolved (e.g., naturally or through different anthropogenic land uses and management practices), (ii) how these are changing over short to long timeframes, and (iii) how future landscapes might appear or be shaped. The latter is particularly pertinent given large predicted impacts of long‐term climate change on global landscapes (Collins et al., 2013), increases in global population and excessive use of natural resources (IPCC, 2019). The importance of obtaining knowledge on land cover and monitoring land cover change has therefore become more evident, particularly given recent emphasis on the need to sustainably use land and ensure resilience for ecosystems and their components, including carbon, water and biodiversity (Henry et al., 2019; Olsson et al., 2019).

Despite common and everyday use, numerous and often disparate descriptions of land cover change have been used for decades, with many being legacy terms that often provide insufficient detail and/or consistency. This lack of harmonized definitions and usage of terms interrupts the chain from data to information, knowledge and wisdom, and limits clarity and hence communication. In turn, local to global efforts aimed particularly at preventing further damage to both natural, and anthropic ecosystems are compromised. These efforts include the design, development and implementation of policies and planning strategies targeting avoidance of ecosystem loss or degradation via activities that focus on conservation, protection, restoration, recovery and/or sustainable land use (Cowie et al., 2018; Herrick et al., 2019). This legacy of incoherent terminology further hinders detection, mapping and monitoring of landscape change processes (Gibbs & Salmon, 2015; Yang et al., 2017), including through Earth observations (EO; Sims et al., 2019). For these reasons, a change framework needs to include a detailed and standardized approach to the classification of land cover dynamics that is relevant across different spatial and temporal scales; allows upscaling and downscaling; meets the criteria of detail needed for granular decisions by local land managers and gives flexibility across domain applications (e.g., land restoration for protection of nature, climate mitigation actions, avoiding further biodiversity loss, etc.).

The aim of this research, therefore, was to develop and explain a new globally relevant and scalable change framework that included (i) a Global Change Taxonomy, with this providing consistent descriptions of past, present and future land cover change whether observed on the ground or remotely (from airborne and spaceborne sensors) or predicted (e.g., from process‐based and/or distribution models), and (ii) an associated Evidence‐Based Change Framework to support decisions regarding the types and causes of changes listed in the Global Change Taxonomy. The proposed framework builds on a combination of structured land cover taxonomies, using the globally applicable United Nation's (UN) Food and Agriculture Organization (FAO) Land Cover Classification System (LCCS) (Di Gregorio & Jansen, 2000) as demonstration, and the Driver‐Pressure‐State‐Impact‐Response (DPSIR) Framework (Oesterwind et al., 2016). The LCCS framework describes states while the DPSIR Framework uses information on states; thus, their combination was exploited to underpin the Global Change Taxonomy and facilitate consistent, standardized and detailed descriptions of change based on evidence. In particular, knowledge of the changing states of landscapes (e.g., vegetation structures and compositions, water quality) can be accumulated within the Evidence‐Based Change Framework to discern the impacts, with evidence from the same or similar sources used to identify pressures leading to change. Both the LCCS and DPSIR are also recognized at national and international levels and, respectively, have been widely used to support decisions (e.g., policy, land management) and inform land cover mapping and monitoring.

The change framework has been tailored for use with EO data and within associated processing infrastructures (e.g., big data computing). As such, it complements and supports existing efforts aimed at detecting and describing change (e.g., in land cover and use), including those utilizing long time‐series of EO data and derived metrics (e.g., for disturbance detections or trend analyses). The framework has been developed so it can inform modelling of past and/or future landscapes (e.g., vegetation), ecosystems (e.g., biodiversity, function) and environments (e.g., climate), either singularly or in combination. This includes use of integrated environmental assessment models (Laniak et al., 2013), intermediate climate models (e.g., Joshi et al., 2014) or dynamic vegetation models (Daniel et al., 2016), within which cause–effect relationships and transition probabilities respectively can be better described, quantified and integrated. While elaborate, the framework purposely uses concepts and terms that have been developed to facilitate understanding by a broad range of stakeholders and encourage wide uptake and use across multiple domains.

## 2 BACKGROUND

### 2.1 Land cover taxonomies

A wide range of taxonomies have been developed for use across multiple scales to describe and map land covers. At the sub‐national level, classes defined (and associated legends) are often specific to the local area and/or conditions (e.g., Preidl et al., 2020), but these become more generic and less detailed for national (country‐level) classifications. An example is the taxonomy of Anderson et al. (1976), which has 20 classes and forms the basis of the US National Land Cover Database (NLCD) for the United States (Wickham et al., 2021; Yang et al., 2017). For regional descriptions, several relevant taxonomies are often combined, as in the case of the European Space Agency's (ESA) Climate Change Initiative (CCI) Land Cover map of Africa, which integrates elements of several schemes including Globeland30 (Chen et al., 2015) and AFRICOVER (Kalensky, 1998). At the global level, legends have been designed to represent common and widely distributed landscapes and are often necessarily broad and less detailed. Examples include the International Geosphere Biosphere Program's (IGBP) DISCover (Belward et al., 1999) taxonomy, with 17 classes, and the ESA CCI Land Cover (2017) product with 22 classes. In most cases, the resulting land cover products have a ‘flat’ structure in that taxonomic classes are wholly classified. For example, Dabija et al. (2021) uses the classes ‘broadleaved forests’ and ‘coniferous forests’, as defined by the European Corine Land Cover (CLC) categories. Other taxonomies are more structured, including EAGLE (Arnold et al., 2016) and the FAO LCCS. In many cases, these structured taxonomies are not used to their full potential as only subsets of the available classes are typically referenced, primarily to keep taxonomic legends manageable and easy to understand. For example, the ESA CCI Land Cover (2017) uses a pre‐defined subset of FAO LCCS end classes (e.g., tree cover, broadleaved, evergreen, closed to open [>15%], cropland, irrigated or post‐flooding). Therefore, these taxonomies are generally underutilized, as they have been designed to provide far more detailed and in‐depth descriptions of land cover. Coincidentally, they are also well suited for describing and building evidence for changes in the landscape, particularly as the different landscape components can be treated separately, although they have rarely been adopted for this purpose. For this study, the FAO LCCS (Version 2; Di Gregorio, 2005) was used to showcase the Global Change Taxonomy and to decipher change through the Evidence‐Based Change Framework, although other similarly structured taxonomies could be exploited. The advantage of the LCCS is that it is globally applicable and provides a complete, mutually exclusive and hierarchical approach to classification that captures characteristics of the land covers on the ground but is not driven by or restricted to EO‐derived information only.

### 2.2 The DPSIR framework

**FIGURE 1:**
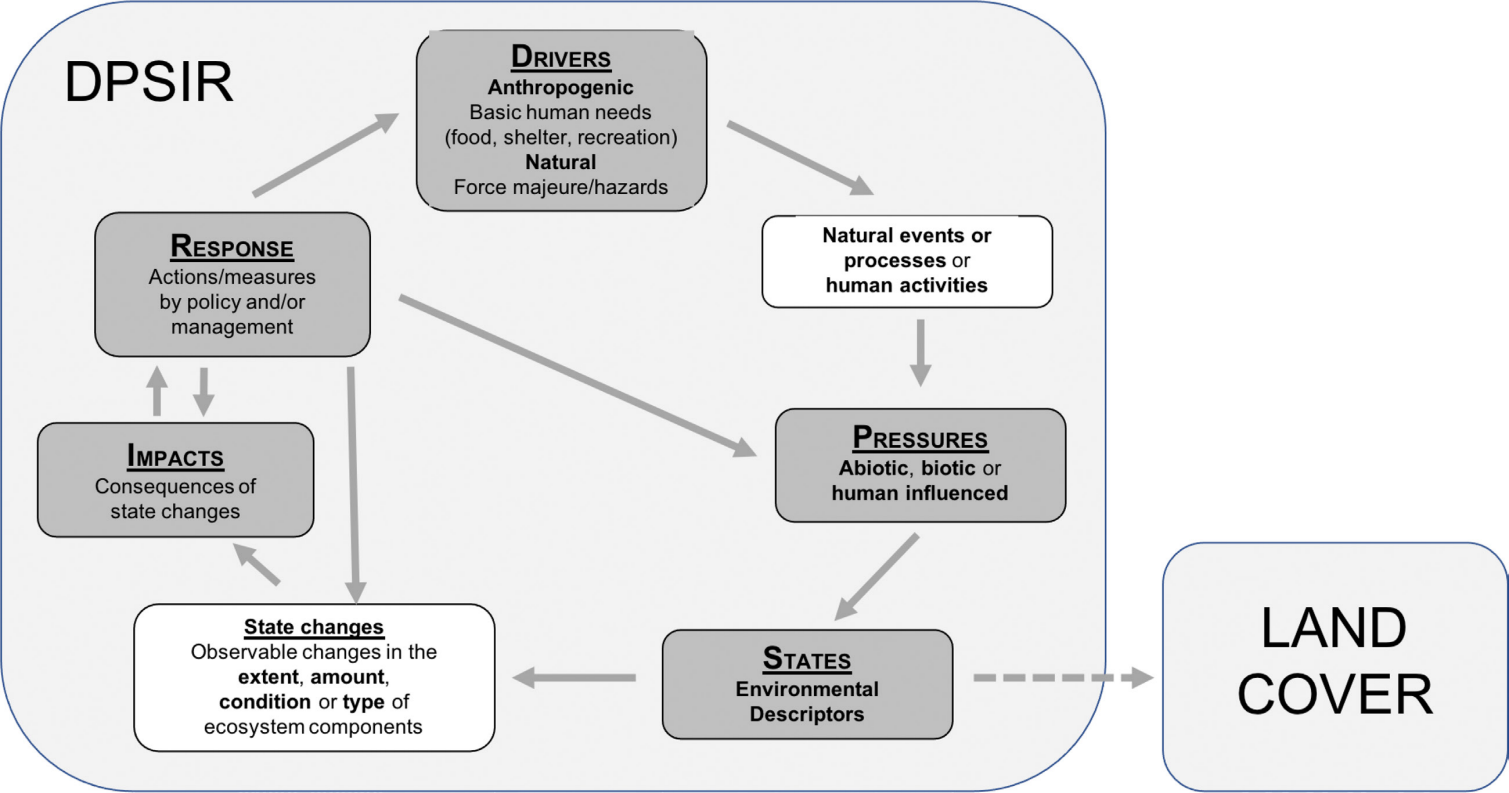
The Driver‐Pressure‐State‐Impact‐Response (DPSIR) Framework (modified from OECD, 1993). The solid arrows indicate where one DPSIR component potentially causes changes in another. The dotted arrow indicates that land cover is a measure of the environmental state at a given location and time.

The DPSIR Framework (Figure 1) was developed by the European Environment Agency (EEA) from the Pressure‐State‐Response (PSR) framework (OECD, 1993) to support State of the Environment reporting, bridge gaps between science and policy, and facilitate understanding of causalities of change. The Framework is described by Oesterwind et al. (2016) and Elliott et al. (2017) and summarized as follows. Drivers are underlying anthropogenic and non‐anthropogenic forces (e.g., increasing atmospheric CO_2_, demand for resources) that generate pressures on the environment. The main drivers of change are abiotic, biotic or anthropogenic. Abiotic drivers are associated primarily with climate and include changes in precipitation and land and sea temperatures (with these influencing flood, snow and fire regimes and levels of oceans and ground waters); geological activity (e.g., volcanos, earthquakes); and solar and lunar cycles (influencing day lengths and tides, respectively). Biotic drivers relate to changes in the distribution, abundance and/or types of flora and fauna. Anthropogenic drivers are more varied, with examples including economic activity, wars, changes in population, or the social desire and/or need to conserve, protect or restore the environment. Drivers are the primary causes of pressures.

Pressures result in changes in environmental states. Pressures can arise from natural events (e.g., extreme weather events, wildfires, insect infestations), natural processes (e.g., long‐term droughts, sea level fluctuations resulting in a chemical intrusion disturbance) or human activities (e.g., ploughing, crop rotation, mining, drainage). Each has the potential to change all or part of the state(s) of the environment (e.g., anthropogenic landscapes, habitats, ecosystems). Pressures are, in effect, the reasons for observed changes in the state, measured by physical, chemical and/or biological characteristics of the environment. Pressures can exert adverse (negative) or positive influences on the ecosystem as a whole, or on different ecosystem components (e.g., vegetation, soil, water, air) in relation to the functions they fulfil. Pressures can also be introduced (e.g., through policy, practice) to enact change.

Impacts are the consequences of individual or collective changes in state (e.g., in one or more attributes relating to vegetation structure, floristics or biomass, water type and/or quality, urban configurations, termed herein as Environmental Descriptors [EDs]). Here, we consider only impacts on the environment and ecosystems, though changes in state can also result in impacts on the economy, society and human well‐being.

Changes in state can be quantified in terms of the extent, amounts or types of EDs, which describe the impact. Changes in amount can also be further differentiated according to whether these are extensive or intensive (Scheider & Huisjes, 2019). In the context of land cover, an extensive property is an amount that is additive and proportional to the size of the system, with examples being vegetation biomass (Mg ha^−1^), canopy cover (%), height (m) and Leaf Area Index (LAI, m^2^ m^−1^) or water velocity (m s^−1^). An intensive property is an amount (or quantity) where the value is independent of the amount of a substance or the extent of a system and is not necessarily homogeneously distributed in space. Examples are water or land temperature (°C), air pressure (pascal units, pa) and chemical concentration (μmol m^−2^). Changes in intensive properties (e.g., foliar chemistry) might occur even if the extensive property (e.g., canopy cover; %) remains the same. These can also vary in unison, with an example being a decrease in both water depth (m) and temperature (°C), an increase in both canopy cover (%) and chlorophyll concentrations (μmol m^−2^), or an increase in the depth of sediment deposited (m) but a decrease in average grain size (cm). Most changes (in amount or type) reflect a perceived deterioration or improvement in, for example, biological health or water or air quality that is often collectively referred to as change in environmental condition (measured in terms of its physical, chemical and biological characteristics) or ecosystem condition (additionally measuring characteristics related to the processes and interactions that connect living organisms with each other and non‐living components and influence their function). Condition is a collective term for quality that is benchmarked against a pre‐determined goal or target and helps inform the level of concern implied by an impact on (i.e., a change in) the amount, extent and/or type of something, and is usually derived from a number of indicators rather than a single measure of change. Integrity is also used as an alternative term for condition (Nicholson et al., 2021).

The extents, amounts (intensive and extensive) or types of components (land, water or atmosphere) can be interpreted qualitatively or quantitatively (e.g., through on‐ground or remote measurement). An example is vegetation dieback, which is an impact resulting from multiple pressures that could be abiotic (e.g., drought, storms or sea level fluctuations), biotic (e.g., pathogens, herbivory by insects or mammals) or anthropogenic (e.g., pollution, mooring/anchoring disrupting sea grass beds). Evidence for this impact can be gathered from knowledge of changes in one or more characteristics of states (e.g., EDs reflecting amounts of canopy cover, dead plant material, and/or foliar chemistry or species type). When benchmarked against ideal states, these changes in states can inform interpretation of environmental and/or ecosystem conditions as increasingly departing from or approaching this ideal. Pressures leading to impacts may be singular (e.g., rising sea levels) or multiple (e.g., intense storms or drought as well as rising sea levels), with each ranging in magnitude from being subtle and often difficult to discern (e.g., herbivory by insects) to substantive and actually or potentially irreversible (e.g., vegetation loss because of deforestation).

An impact resulting from one or more pressures may trigger a human response (an action), often mediated through policy or land management decisions, which is intended to address, reduce or prevent unwanted (negative) changes or develop a positive change in the environment. Often, these responses lead to additional pressures that initiate further changes in states and, in turn, cause impacts and potentially further responses. The magnitude of the impact and the level of concern (i.e., through an interpretation of condition) often determines the nature of the response. For example, dieback of 5% of trees in commercial plantations might trigger increased monitoring to establish whether pathogens might be spreading, >30% might lead to actions to avoid further spread within the plantation or beyond, including selective removal and disposal of infected trees, while >90% might force clear cutting of both infected and non‐infected plantations of the same species. Therefore, policy and land management decisions are often responsive to the magnitude (as well as the associated uncertainty) of changes, particularly if these lead to a significant alterations in the extent or overall condition of environments or ecosystems and have an adverse impact on humans and/or nature.

## 3 CONCEPTS BEHIND THE GLOBAL CHANGE TAXONOMY

### 3.1 Land cover class construction using environmental descriptors

**FIGURE 2:**
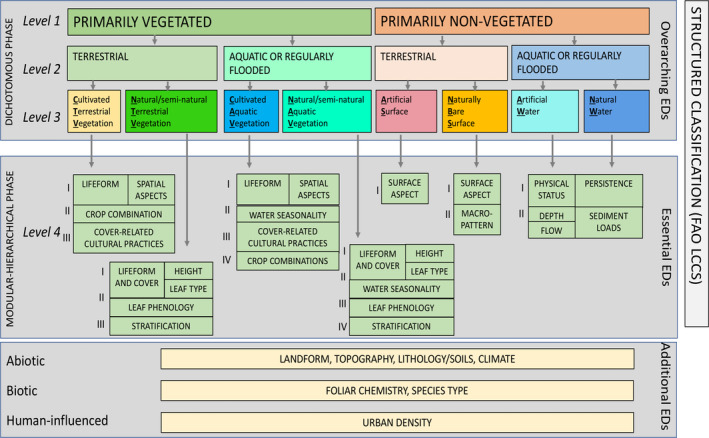
Overview of the FAO LCCS, highlighting the dichotomous (to Levels 1, 2 and 3) and the modular‐hierarchical phases, with progressive steps in the hierarchy indicated by roman numerals (I–IV). Modified from Di Gregorio (2005). FAO, Food and Agriculture Organization; LCCS, Land Cover Classification System.

Hierarchical (structured) taxonomies developed for land cover classification have an established set of classes that provide the initial broad divisions of the dominant cover (e.g., croplands, urban, water). These can be termed Overarching Environmental Descriptors (OEDs). As an example, the dichotomous phase of the LCCS (Figure 2) first identifies primarily vegetated and aquatic or regularly flooded land covers and their opposites (non‐vegetated and terrestrial) at Levels 1 and 2 respectively. These are then cross tabulated with those defined as natural or otherwise (i.e., cultivated/managed or artificial) to generate eight overarching land cover classes at the third level (referred to as Level 3). OEDs representing vegetated and aquatic land covers can be interpreted in the field or mapped (e.g., by thresholding summaries of satellite‐derived vegetation cover fraction [%] or water extent, as determined from temporal observations of water occurrence [frequency over time]). Artificial surfaces (ASs) or cultivated land can be classified directly using, for example, machine‐learning algorithms (Owers et al., 2021).

More detailed descriptions can then be provided within structured land cover taxonomies by referencing EDs specific to each OED. Herein, these are referred to as Essential Environmental Descriptors (EEDs) as they are needed to define and deliver classifications according to the taxonomy used, either partially or in full. Continuing with the LCCS as the example, the modular‐hierarchical phase (Figure 2), termed Level 4 by Lucas et al. (2019) and Owers et al. (2021), includes sub‐levels (I–IV) that successively reference relevant sets of EDs and combine these to provide the final land cover class. Once constructed, further detail can again be added by integrating relevant Additional Environmental Descriptors (AEDs). These are external to the land cover taxonomy and play no part in its formal construction. Examples relevant to the LCCS vegetated classes include above‐ground biomass (Mg ha^−1^) and dominant plant species (numerical codes); for water classes, these might include acidity (pH) or velocity (m s^−1^). Temperature (°C) is an example of an AED that is relevant to all land and water classes. These can be summarized according to whether they are abiotic (e.g., topography), biotic (e.g., foliar chemistry) or human‐influenced (e.g., urban density).

To ensure consistency in the construction and description of land cover classes and scalability of observations, measurements and maps across both space and time (and from local to planetary levels), EDs need to be continuous or categorical and assigned, respectively, defined and measurable units or pre‐defined codes. Conventions for categorical codes are less common, although in many land cover taxonomies, numeric codes for different descriptions of the environment are stated (e.g., those specific to different leaf types, water states or water hydro‐periods within the LCCS; Owers et al., 2021). Consistent code sets have also been put in place for other categories such as plant species (e.g., Turland et al., 2018). Several of these categories can be classified directly while others are generated from summaries of continuous measures relating to the physical, biological or chemical attributes of landscape components, with examples being vegetation canopy cover (%) or annual snow hydroperiod (months) (Lucas et al., 2019). As EDs can be used across scales, so too can land cover classes constructed from these. Both are then relevant, whether described on the ground, remotely or through predictions. EDs are also the equivalent of state indicators in the DPSIR framework.

### 3.2 Environmental descriptors and land cover change

Most field‐based assessments of land cover change are based on personal observations and perceptions and are generally interpreted as a transition from one broad land cover class to another, leading to a change in the extent of these classes. This is termed a land cover conversion. If the class remains the same, a net gain or loss in amounts (intensive and/or extensive) or types of individual components (or combinations of these) can occur, with these reflecting a modification of the land cover. The three terms (extent, amounts and type) collectively provide an overall measure of condition (noting that contextual information may be needed) and can be used to describe a landscape before and after a change from one land cover to another. Extent changes are often conveyed using a transition matrix, which compares their distributions or occurrence between any two time‐separated periods.

Changes in extent might include the transition from natural terrestrial vegetated to an AS, with an example being deforestation for subsequent establishment of urban areas. Such changes (conversions) can be perceived as both a loss of vegetation and a gain in AS, and both viewpoints need to be considered. Amount changes (modifications) include an increase or decrease in vegetation canopy cover (%), water or snow depth (m) or water/soil acidity (pH). Categories associated with type changes include plant species or water state (e.g., snow to water) and are typically assigned alphabetic or numerical (integer) codes and can reflect both conversions and modifications.

Observations of change between any two time‐separated periods (i.e., *T*_1_ and *T*_2_) can be defined through the combinations of OEDs, EEDs and/or AEDs, although this generates many thousands of classes. These are time‐independent if predefined units of measurement or type categories are used and are relevant whether compared minutes or centuries apart. For example, even being different water states, comparisons of water or snow depth (m) will always result in a depth change regardless of the time interval between measurements. Hence, comparisons of EDs and the derived classifications can be used to describe both past and predicted (future) changes. In the latter case, use can be made of individual or combinations of EDs predicted from, for example, (semi‐)empirical, process, species distribution, or state and transition models that consider and encapsulate well‐studied biological, physical and/or chemical phenomena.

## 4 MATERIALS AND METHODS

### 4.1 Describing land cover conversions

**FIGURE 3:**
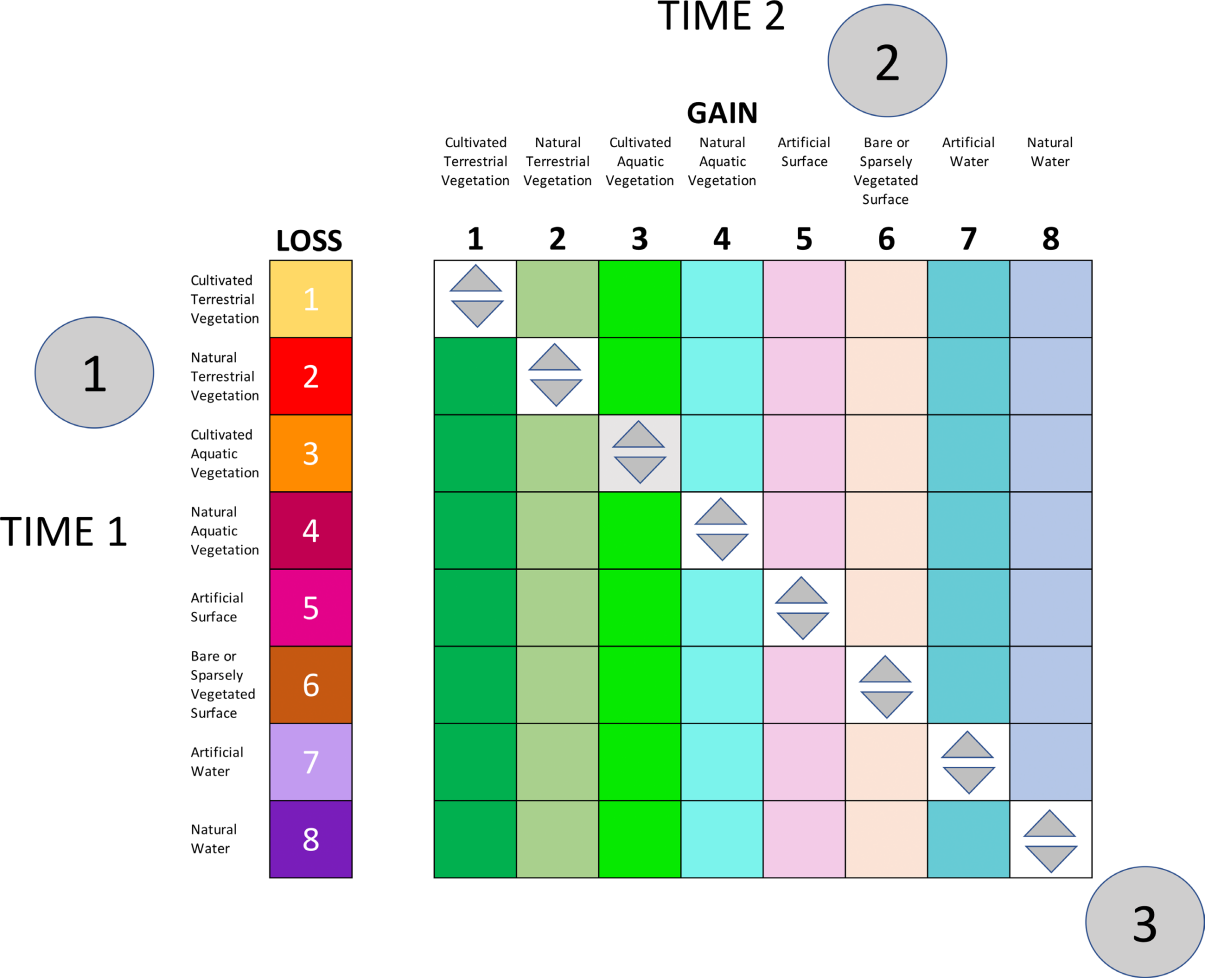
The base transition matrix indicating changes in broad land cover classes (OEDs) of the FAO LCCS, which are comprised of 64 potential classes (56 off‐diagonals, 8 on‐diagonals). The coloured squares summarize 16 broader categories that represent a loss (of eight classes) or gain (of eight classes) in extent at the expense of another. The on‐diagonals nominally indicate no change in extent but a decrease and/or increase in amounts (extensive and/or intensive) of EDs (represented by arrows). A change in type can also occur but being categorical, there is no objective numerical direction. Circled numbers give indicative examples of a loss in extent of (semi‐) natural terrestrial vegetation (NTV) (1) and a corresponding gain in the extent of a bare or sparsely vegetated surfaces (NBS) (2). Increases and/or decreases in amount or changes in type are indicated in (3; all on‐diagonals). EDs, Environmental Descriptors; FAO, Food and Agriculture Organization; LCCS, Land Cover Classification System; OEDs, Overarching Environmental Descriptors.

A first step towards developing a globally relevant and standardized taxonomy and framework for consistently describing land cover change was to establish the transition matrix between observed broad land cover classes (i.e., OEDs). This stage was developed and is illustrated using the FAO LCCS given the dichotomous and then hierarchical modular structure of this taxonomy (Figure 3). The between‐class transitions and within‐class changes were identified by comparing these OEDs (i.e., the eight FAO LCCS Level 3 classes) between any two time‐separated periods (i.e., *T*_1_ and *T*_2_), leading to 64 potential change categories; 56 on the off‐diagonals and 8 on the on‐diagonals. Further auxiliary descriptions were then generated subsequently by comparing the EEDs associated with the FAO LCCS Level 4 categories, with these augmented subsequently using AEDs as outlined below.

#### 4.1.1 Between‐class changes: land cover conversions

Transitions between any two OEDs (off‐diagonal in Figure 3) corresponded to a change in *extent* in both the original class at the time of the first observation (*T*_1_) and the replacement class in the second observation (*T*_2_) (i.e., a land cover conversion). More detailed descriptions of the land cover class prior to and following the change can be provided by referencing the categorical and continuous EEDs used in the construction of the FAO LCCS classes at *T*_1_ and *T*_2_ respectively as well as AEDs. As an example, natural terrestrial vegetation (herein referred to as NTV; and including semi‐natural vegetation) at *T*_1_ could be described according to lifeform (e.g., woody trees or shrubs or herbaceous graminoids or forbs), with each then attributed with information on their heights (m), covers (%), leaf types (codes) and/or phenology (time; summarized to codes representing evergreen, semi‐evergreen, deciduous or mixed woody or annual or perennial herbaceous lifeforms). However, if this vegetated land cover was converted to a naturally bare or sparsely vegetated surface (NBS), the resulting class at *T*_2_ could then be described based on the types, sizes and consolidation characteristics of bare surface materials (again defined by the LCCS). Therefore, a change in extent of the OEDs can affect all or many of the environmental descriptors (EEDs, and AEDs) of the original and replacement land covers, with some or all components added or removed.

#### 4.1.2 Within‐cover changes: land cover modifications

Where the OED class remains the same between time‐separated periods (the on‐diagonals in Figure 3), a modification rather than conversion of the land cover occurs and only changes in the amounts or type of state indictors within the OED can take place. These changes can only be described by considering variations (decreases, increases or no difference) in EED or AED units of measurements (or categorizations of these) or simply a change in a pre‐defined category (e.g., representing lifeform or leaf type). For example, where no change in the class NTV is observed between *T*_1_ and *T*_2_, the vegetation lifeform could still change from woody to herbaceous (although only within or between each of the vegetated classes; i.e., NTV, natural aquatic vegetation [NAV], cultivated terrestrial vegetation [CTV] or cultivated aquatic vegetation [CAV]), as can vegetation amounts (e.g., canopy cover, plant height or foliar chemical concentrations) and type (e.g., dominant plant species). The same applies for other OEDs, such as water and AS, where changes in relevant EEDs and AEDs would be observed or quantified.

### 4.2 Observed changes in land cover—an essential initial assessment

Whether on the ground or from a remote viewpoint, either between‐class or within‐class changes in land cover can be observed and interpreted in several ways. For example, the conversion of NTV to an AS could be viewed as either (i) a loss of vegetation extent or (ii) a gain in urban area. Conversely, while remaining as natural water (NW), areas might be observed as experiencing an increase or decrease in extensive amounts of water depth and/or intensive amounts of water velocity or turbidity, and/or a change from a liquid to a frozen water state (type) as a result of temperature changes. While changes in amount can be described as directional (positive or negative, depending on context), changes in type generally cannot. Interpretations of directions of change are therefore often user‐specific (e.g., from woody to herbaceous lifeforms or deep to shallow water) and may be construed as either positive or negative depending on the situation and context.

For the reasons above, a distinction was made between observed losses and gains in the extents of the OED classes, which can be considered or represented separately (e.g., as individual spatial layers representing either losses or gains). Where the OED class remained the same, the distinction was made between observed decreases and/or increases in amounts. Changes in type were simply registered as a change, thereby allowing the user to decide the importance and implications. Once these observed changes had been described (and judged to be appropriate for mapping), they provided the basis for building the Global Change Taxonomy and associated glossary and defining the evidence‐base needed to support assignment to specific change classes.

A comparison of the eight classes of the LCCS Level 3 identified 24 broad and commonly observed changes. Of these, 16 represented a *change in extent* (i.e., gains or losses; off‐diagonals). By way of illustration, the NTV extent may decrease (Circled 1; in Figure 3) and be replaced by up to seven Level 3 (OED) categories (i.e., CTV, CAV, NAV, AS, NBS, AW and NW). Correspondingly, an observed gain in extent for up to seven of these categories (e.g., NBS; Circled 2) can occur. Where a change in extent was indicated, the amounts and type of the component EDs of each land cover prior to and following a change could be described. In this case, and as indicated, the EEDs and AEDs used often differed before and after the change. For example, after a flood, NTV of a certain lifeform (e.g., herbaceous), height, cover and mix of annual/perennial forbs and graminoids could be replaced with water that might be deep, flowing and turbid. A further eight categories, located on the on‐diagonals (Circled 3; in Figure 3), were indicative of no change in extent.

### 4.3 Defining and providing evidence for change

#### 4.3.1 Combining pressures and impacts to describe change

In developing the Global Change Taxonomy, the combination of impacts and pressures defined in the DPSIR was recognized as providing overarching descriptions of many of the changes occurring globally. On this basis, the framework of the taxonomy was designed to logically link different impact and pressure terms and build evidence for these by combining information on changes in state indicators (i.e., EDs). The notation ‘impacts (pressures)’ was adopted to describe both the consequence of an observed change and its associated reason(s).

On this basis, the categories of the Global Change Taxonomy were generated by first listing types of impacts commonly observed globally and confirming their relevance and applicability across scales (e.g., national to local). The pressures leading to these were then reviewed. For both impact and pressure terms, a search of the literature and web‐based materials was conducted to provide standardized definitions for each. These were then reviewed by the authors and other experts and consolidated in a glossary.

#### 4.3.2 Gathering evidence for impacts and pressures from EDs

**FIGURE 4:**
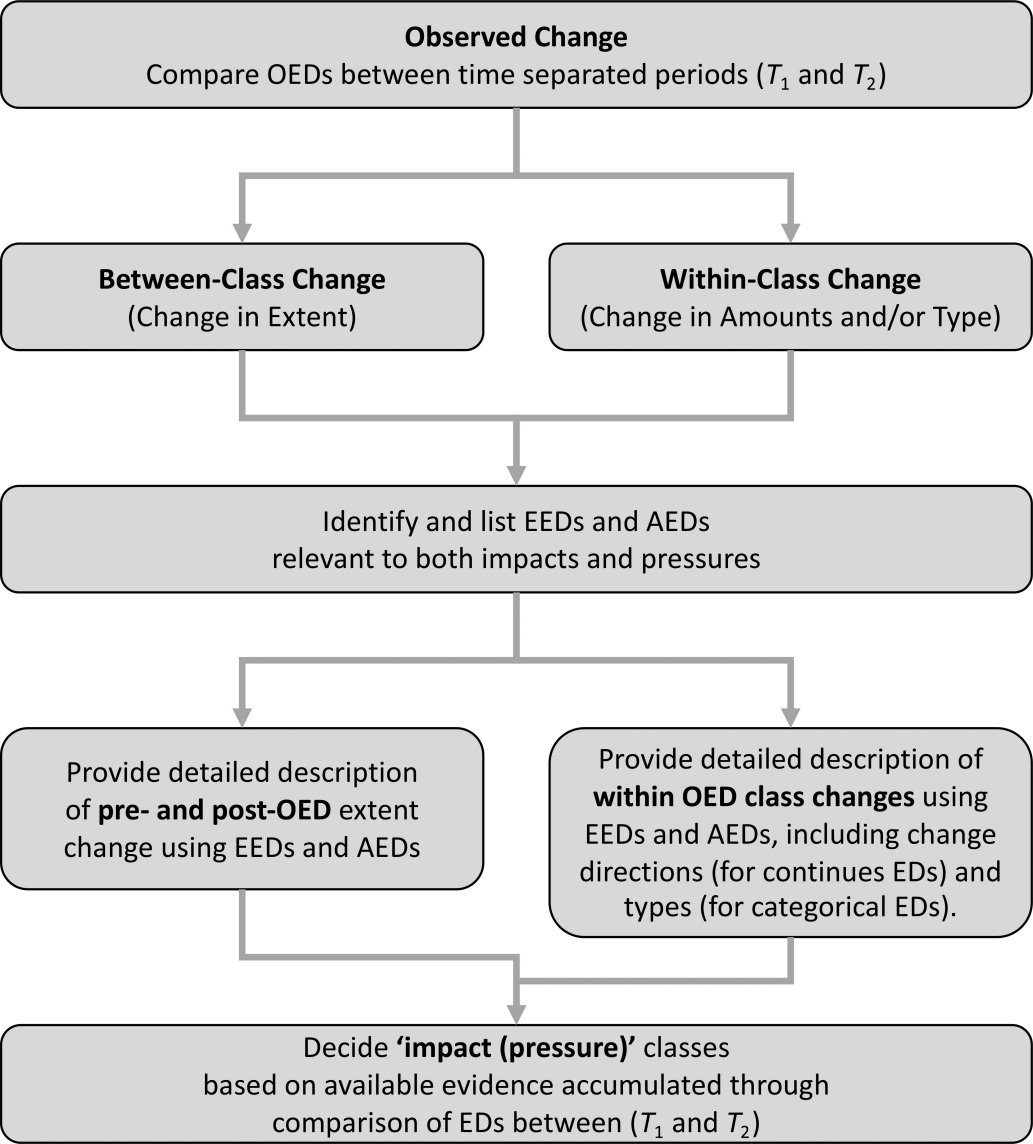
An overview of steps taken to first characterize observed change and then differentiate impacts and pressures through accumulation of evidence on changes in extent (for between class changes) and amounts and type (for within class changes) from relevant and available sources (ground, remote or modelled).

For each of the impact and pressures listed in the Global Change Taxonomy, the EDs (OEDs, EEDs and AEDs) needed to provide evidence for each, and hence the different ‘impact (pressure)’ change categories, were also reviewed. A stepwise approach was undertaken to develop the evidence base (Figure 4), as follows:

- The 24 observed changes in land cover classes were described by comparing the OEDs (in this case, the eight FAO Level 3 categories), representing eight losses, eight gains and eight no‐class change combinations between two time‐separated periods (*T*_1_ and *T*_2_), with this providing the first line of evidence. These changes reflected broad losses or gains in (i) extent or (ii) amounts and/or type. Specific conclusions about impacts and pressures cannot generally be drawn from these because of insufficient evidence at this stage.
- The individual EDs that could contribute further evidence for the impacts and/or pressures were then identified and listed. These were comprised primarily of EEDs and AEDs, noting that these often differed between impact and pressure categories.
- Where the OEDs changed between *T*_1_ and *T*_2_, these took precedence and EEDs and AEDs were then only used to describe the land cover prior to and after a natural event or process or human activity. However, where these remained the same, the EEDs and AEDs (relating to amounts or types) provided the primary source of evidence for within‐class changes.
- For continuous EDs associated with each of the impact and pressure combinations (e.g., canopy cover, water turbidity), the general direction of change (loss or gain in extent or an increase/decrease in amounts) was expressed using a – or + symbol, noting that a continuum of change in magnitude is common in many situations. For categorical EDs (e.g., representing lifeform, species type), only whether a change might take place could be indicated (represented by a single simple Δ) as lettered or numbered codes are non‐directional and open to interpretation. Some changes can, however, be construed as negative or positive depending on particular situations or user perspectives.
- From this information, user‐defined and knowledge‐based rules were constructed using domain‐specific EDs accumulated through the evidence‐base to discern the ‘impact (pressure)’ category.

In cases where the available evidence might be insufficient to conclude the allocation to an ‘impact (pressure)’ class, several might be assigned, with ‘dieback (pathogens)’ or ‘dieback (drought)’ being an example. This step then serves to identify gaps in knowledge or data, promote the development of new or improved methods for retrieval or classification of EDs, or explore substitute information sources.

As an illustration of the use of the evidence‐base, an observed transition from NTV to a NBS indicates vegetation loss (extent) (the impact), which might be the result of several pressures, including deforestation, wildfire or vegetation clearance (that may act in isolation or in combination). However, where the NTV woody vegetation class is observed as remaining the same but vegetation amounts (extensive and/or intensive) decrease, this could be attributed to a different set of pressures, including selective logging, thinning and/or prescribed burning. In each case, further evidence needs to be accumulated to allow the reasons for change (i.e., the specific pressure(s)) to be distinguished. A distinction was made also between evidence that was needed to make the decision on the allocation of a change category (required evidence) and that which affirms this decision (confirmatory evidence). From this review, an evidence matrix for all listed ‘impact (pressure)’ combinations was developed to support, oppose or change decisions regarding allocation to each of these, noting that this is open to amendment based (for example) on user knowledge and expertise.

### 4.4 The importance of time

The evidence needed to support the allocation of change to different ‘impact (pressure)’ categories of the Global Change Taxonomy was scale independent given the requirement for all inputs to be quantified or classified according to defined units or categories respectively. However, the ability to detect the reasons for change was found to have some time‐dependence and there was often alignment with definitions and terms that inherently inferred a time component (e.g., flash flood, ploughing and deglaciation). The time dimension was therefore included as additional evidence to interpret the nature of change, with this giving consideration to the time span of the actual natural event or process or human activity (occurrence), the time between commencement and detection (lag) and the time period of detectability (manifestation), whether from ground or remote observations, and the time from commencement to completion (duration), whether actual or perceived. The duration is often the most difficult to quantify, particularly as it can be associated with the time taken to recover to a previous, desirable or reference state, but can also be well defined (e.g., as in the case of ploughing or clear felling of diseased forests, the durations of which have clear bounds).

The following serve as two illustrations of these descriptors of time. Strong winds (the pressure) associated with a storm event may persist over a period of several hours (the occurrence; no lag) and cause widespread vegetation damage (the impact), which might be observed for years after (manifestation). Plant pathogens (the pressure) may establish and be present for several months or even years before vegetation dieback (impact) becomes evident (the lag), but this impact may be continue to be observed (the manifestation) over several decades as loss occurs through progressive reductions in leaf cover and decomposition of standing and fallen wood. The periods of occurrence, lag and manifestation can vary from the beginning of a natural event or process or human activity to the end of its impact. This can often be longer than the manifestation period, which therefore justifies the need for including and using the full duration. The assignment of these time‐dependent categories to impacts and pressures and their combinations were based on knowledge supported by scientific literature reviews.

The time components of occurrence, lag, manifestation and duration were included in the evidence‐base, which simply asked: how long does the change event, process or activity take? (occurrence), how long before the change can be seen? (lag), how long can the change be seen for? (manifestation), and how long is the period from when the change is first detected to when it ends or transitions to another impact/pressure category. The periods of time considered were divided into the broad classes of sub‐daily to daily, weeks (up to 3 months), seasonal (typically 4–6 months), annual, multi‐annual, decadal and centuries, noting that overlaps between these groupings can and often occur. The time(s) since the last natural event or process (e.g., ‘vegetation loss (amount) (bushfires)’, ‘vegetation dieback (drought)’ or human activity (‘vegetation loss (extent) deforestation’)) and the frequency of occurrence and time interval between these can also be considered as descriptors of change, though these are more emergent properties of particular ‘regimes’ of change.

## 5 RESULTS

### 5.1 The Global Change Taxonomy and glossary

Based on the review and expert knowledge, 144 pressures (reasons behind a change; Table S1) and 77 impacts of change (consequences) were identified (Table S2). The impact classes related to the main OED categories of cultivation (agriculture; 8), natural or semi‐natural vegetation (19), AS (urban; 14), naturally bare or sparsely vegetated surfaces (10) and natural or artificial water (26), as defined by the FAO LCCS but noting that these are common to other structured classifications. The taxonomy considered abiotic, biotic and anthropogenic drivers of change and of the 144 pressures listed, 71 were linked to natural events or processes while 73 were associated with anthropogenic activities.

When the impacts were linked to pressures, 246 combined classes were identified (listed in Table 1). Examples associated with abiotic, biotic and anthropogenic pressures respectively included ‘crop damage (increased wind)’, ‘snow accumulation (snowfall)’ and ‘elevation change (subsidence)’; ‘vegetation gain (extent) (colonization)’ and ‘vegetation species change (succession)’; and ‘railway or road abandonment (reduced investment)’, ‘vegetation loss (extent) (deforestation)’ and ‘water gain (extent) (wetland restoration and/or construction)’.

**TABLE 1:** List of combined impacts and pressures used to describe change.

Impact (pressure)	Impact (pressure)
Accretion (sediment transport)	2 Vegetation dieback (cold snap)
3 Algal bloom (eutrophication)	4 Vegetation dieback (drought)
5 Algal bloom (high inland water temperatures)	6 Vegetation dieback (heatwave)
7 Algal bloom (increased temperature)	8 Vegetation dieback (non‐insect herbivory [natural])
9 Algal dieback (decreased temperature)	10 Vegetation dieback (increased wind)
11 Bare soil exposure (burning)	12 Vegetation dieback (pathogens)
13 Bare soil exposure (erosion)	14 Vegetation dieback (pollution)
15 Bare soil exposure (ploughing)	16 Vegetation dieback (prolonged inundation)
17 Bare soil exposure (tillage)	18 Vegetation dieback (prolonged snow cover)
19 Blackwater event (inundation following extended drought)	20 Vegetation dieback (water salinisation)
21 Vegetation Browning (decreased precipitation)	22 Vegetation dieback (sea level fluctuation)
23 Building or infrastructure abandonment (dam removal)	24 Vegetation dieback (soil salinisation)
25 Building or infrastructure abandonment (flooding)	26 Vegetation gain (amount) (thinning)
27 Building or infrastructure abandonment (increased wind)	28 Vegetation gain (amount) (afforestation)
29 Building or infrastructure abandonment (urban fire)	30 Vegetation gain (amount) (bushfire recovery)
31 Compaction (increased traffic)	32 Vegetation gain (amount) (ecological restoration)
33 Compaction (overgrazing [stock])	34 Vegetation gain (amount) (encroachment)
35 Compaction (overgrazing [natural])	36 Vegetation gain (amount) (farmland abandonment)
37 Coral bleaching (increased acidity)	38 Vegetation gain (amount) (fertiliser application)
39 Coral damage (pathogens)	40 Vegetation gain (amount) (growth)
41 Coral bleaching (prolonged temperature increase)	42 Vegetation gain (amount) (reduced or cessation of grazing)
43 Coral damage (sedimentation)	44 Vegetation gain (amount) (reforestation (natural)
45 Coral damage (invasive or exotic species)	46 Vegetation gain (amount) (reforestation (plantations)
47 Coral recovery (decreased acidity)	48 Vegetation gain (amount) (regrowth)
49 Coral recovery (prolonged temperature decrease)	50 Vegetation gain (amount) (removal of herbivores)
51 Crop change in cultivated lands (crop rotation)	52 Vegetation gain (amount) (revegetation)
53 Crop damage (drought)	54 Vegetation gain (amount) (urban greening)
55 Crop damage (excess precipitation)	56 Vegetation gain (amount) (vegetation thickening)
57 Crop damage (excess rain)	58 Vegetation gain (extent) (afforestation)
59 Crop damage (flooding)	60 Vegetation gain (extent) (colonisation)
61 Crop damage (grazing [natural])	62 Vegetation gain (extent) (ecological restoration)
63 Crop damage (grazing [stock])	64 Vegetation gain (extent) (greenspace construction)
65 Crop damage (increased wind)	66 Vegetation gain (extent) (mine site rehabilitation)
67 Crop damage (insect herbivory)	68 Vegetation gain (extent) (planting)
69 Crop damage (strong winds)	70 Vegetation gain (extent) (rehabilitation)
71 Crop establishment (planting)	72 Vegetation gain (extent) (revegetation)
73 Cropland gain (agricultural expansion)	74 Vegetation gain (extent) (snowmelt)
75 Cropland gain (farmland creation)	76 Vegetation gain (extent) (decreased wave action)
77 Cropland loss (agricultural loss)	78 Vegetation health deterioration (abandonment of fertilizer application)
79 Cropland loss (animal stock change)	80 Vegetation health deterioration (decreased nutrient supply in soil)
81 Cropland loss (fallowing)	82 Vegetation health improvement (fertiliser application)
83 Cropland loss (farmland abandonment)	84 Vegetation health improvement (increased nutrient supply)
85 Cropland loss (idle or fallow in rotation)	86 Vegetation health improvement (irrigation)
87 Deglaciation (prolonged temperature increase)	88 Vegetation loss (extent) (bushfire)
89 Desalinisation (gypsum application)	90 Vegetation loss (extent) (deforestation)
91 Desertification (prolonged temperature increase)	92 Vegetation loss (extent) (drought)
93 Elevation change (deposition)	94 Vegetation loss (extent) (excess rain)
95 Elevation change (earthquake)	96 Vegetation loss (extent) (farmland abandonment)
97 Elevation change (landslide)	98 Vegetation loss (extent) (flooding)
99 Elevation change (mining)	100 Vegetation loss (extent) (land reclamation)
101 Elevation change (subsidence)	102 Vegetation loss (extent) (sea defence construction)
103 Elevation change (waste dumping)	104 Vegetation loss (extent) (severe thunderstorm)
105 Erosion (construction)	106 Vegetation loss (extent) (strong winds)
107 Erosion (excess precipitation)	108 Vegetation loss (extent) (vegetation clearance)
109 Erosion (frost)	110 Vegetation loss (extent) (wave action)
111 Erosion (increased traffic)	112 Vegetation reduction (amount) (bushfire)
113 Erosion (increased wind)	114 Vegetation reduction (amount) (coppicing)
115 Erosion (sea level fluctuation)	116 Vegetation reduction (amount) (decreased nutrient supply in soil)
117 Erosion (topsoil removal)	118 Vegetation reduction (amount) (farmland abandonment)
119 Erosion (water movement change)	120 Vegetation reduction (amount) (fuelwood collection)
121 Erosion (wave action)	122 Vegetation reduction (amount) (harvesting)
123 Flooding (excess rain)	124 Vegetation reduction (amount) (herbicide/pesticide application)
125 Flooding (excess snow)	126 Vegetation reduction (amount) (non‐insect herbivory [natural])
127 Geomorphological change (mining)	128 Vegetation reduction (amount) (insect herbivory)
129 Glaciation (prolonged temperature decrease)	130 Vegetation reduction (amount) (mowing)
131 Greening (increased precipitation)	132 Vegetation reduction (amount) (overgrazing [stock])
133 Increased sediment load (sediment transport)	134 Vegetation reduction (amount) (overgrazing [natural])
135 Inundation (flooding).	136 Vegetation reduction (amount) (prescribed burn)
137 Inundation (sea level fluctuation)	138 Vegetation reduction (amount) (sedimentation)
139 Lava Flow (volcanic eruption)	140 Vegetation reduction (amount) (selective logging)
141 Leaf scorch (strong winds)	142 Vegetation reduction (amount) (stubble burn)
143 Mine abandonment (reduced investment)	144 Vegetation reduction (amount) (thinning)
145 Mine expansion (increased investment)	146 Vegetation reduction in understory (amount) (non‐insect herbivory [natural])
147 Natural surface gain (deposition)	148 Vegetation reduction in understory (amount) (grazing [natural])
149 Natural surface gain (urban rehabilitation)	150 Vegetation reduction in understory (amount) (grazing [stock])
151 Natural surface loss (mining)	152 Vegetation species change (amenity development)
153 Net snow gain (amount) (snowfall)	154 Vegetation species change (atmospheric deposition)
155 Net snow loss (amount) (snowmelt)	156 Vegetation species change (control of invasive or exotic species)
157 Net snow gain (extent) (snowfall)	158 Vegetation species change (decreased acidity)
159 Net snow loss (extent) (snowmelt)	160 Vegetation species change (decreased alkalinity)
161 Net snow gain (hydroperiod) (prolonged temperature decrease)	162 Vegetation species change (decreased nutrient supply in soil)
163 Net snow loss (hydroperiod) (prolonged temperature increase)	164 Vegetation species change (flooding)
165 Phenological change (natural diurnal and seasonal cycles)	166 Vegetation species change (grazing [stock])
167 Railway or road abandonment (reduced investment)	168 Vegetation species change (grazing [natural])
169 Railway or road construction (increased investment)	170 Vegetation species change (ground water recharge)
171 Receding Flood (reduced runoff post flood)	172 Vegetation species change (ground water extraction)
173 Salinisation (evaporation)	174 Vegetation species change (pesticide application)
175 Salinisation (sea level fluctuation)	176 Vegetation species change (increased acidity)
177 Sea ice decrease (prolonged temperature increase)	178 Vegetation species change (increased alkalinity)
179 Sea ice increase (prolonged temperature decrease)	180 Vegetation species change (increased nutrient supply in soil)
181 Sea level fall (ocean‐atmosphere oscillations)	182 Vegetation species change (invasive/exotic species)
183 Sea level rise (melting ice sheets/glaciers)	184 Vegetation species change (overgrazing [stock])
185 Sea level rise (thermal expansion)	186 Vegetation species change (overgrazing [natural])
187 Sedimentation (dredging)	188 Vegetation species change (pathogens)
189 Sink hole (subsidence)	190 Vegetation species change (prolonged inundation)
191 Snow accumulation (snowfall)	192 Vegetation species change (burning)
193 Snow melt (increased temperature)	194 Vegetation species change (succession)
195 Urban area loss (earthquake)	196 Vegetation species change (undergrazing [stock])
197 Urban area loss (flooding)	198 Vegetation species change (undergrazing [natural])
199 Urban area loss (tropical cyclone)	200 Vegetation species change (pollution)
201 Urban damage (flooding)	202 Water depth decrease (abstraction)
203 Urban damage (increased wind)	204 Water depth decrease (dam failure)
205 Urban damage (urban fire)	206 Water depth decrease (dam removal)
207 Urban decay (dam failure)	208 Water depth decrease (deposition)
209 Urban decay (mine abandonment)	210 Water depth decrease (evaporation)
211 Urban decay (subsidence)	212 Water depth increase (construction)
213 Urban densification (construction)	214 Water depth increase (dredging)
215 Urban development (levelling)	216 Water depth increase (flooding)
217 Urban growth (construction)	218 Water depth increase (sea level fluctuation)
219 Urban renewal (repairing damage)	220 Water depth increase (snowmelt)
221 Urban sprawl (construction)	222 Water gain (extent) (aquaculture expansion)
223 Vegetation damage (bushfire)	224 Water gain (extent) (excess precipitation)
225 Vegetation damage (excess precipitation)	226 Water gain (extent) (flooding)
227 Vegetation damage (excess rain)	228 Water gain (extent) (storm surge)
229 Vegetation damage (flooding)	230 Water gain (extent) (wetland restoration and/or construction).
231 Vegetation damage (frost)	232 Water loss (extent) (aquaculture loss)
233 Vegetation damage (increased wind)	234 Water loss (extent) (drying)
235 Vegetation damage (mechanical intervention)	236 Water loss (extent) (land reclamation)
237 Vegetation damage (prescribed burn)	238 Water loss (extent) (reduced snowfall)
239 Vegetation damage (prolonged snow cover)	240 Water loss (extent) (wetland drainage)
241 Vegetation damage (severe thunderstorm)	242 Water quality change (fracking)
243 Vegetation damage (strong winds)	244 Water quality change (nutrification)
245 Vegetation dieback (anchoring)	246 Water quality change (pollution)

For each impact and pressure term, multiple definitions were available from a range of sources (scientific papers, dictionary or encyclopaedia entries, existing glossaries and/or documents from national or international governments or institutions, e.g., the United States Geological Service [USGS] or FAO). The definitions selected for the glossary were those considered to provide a description that reflected observations on the ground and from remote platforms (satellite, airborne or ground) and sensors. However, the capacity for using several optional definitions was introduced in some cases. The online open glossary with associated terms and definitions is available at 10.5281/zenodo.6884999 and has been designed to enable modifications, enhancements and updates as new information or knowledge become available (e.g., new or refined definitions, feedback from use cases), with this achieved through a system of ongoing peer‐review.

### 5.2 Evidence‐based change

#### 5.2.1 Data sources for impacts and pressures

Evidence sources for impacts and pressures were found to differ. For impacts, the information requirements were directed more towards changes in states, with these occurring either simultaneously or sequentially at specific locations. Evidence for major impacts was first obtained by connecting to the different observed change categories, determined through comparison of OEDs between *T*_1_ and *T*_2_. Where changes in extent were observed, detailed descriptions of the states before (*T*_1_) and after (*T*_2_) were obtained by referring to the full range of EEDs and AEDs relevant to each OED. If no change in the extents of OEDs occurred, evidence for within‐class changes in amounts or types was gathered by referencing both EEDs and selected AEDs within specific domains (e.g., vegetation, agriculture, naturally bare surfaces, urban or water).

**FIGURE 5:**
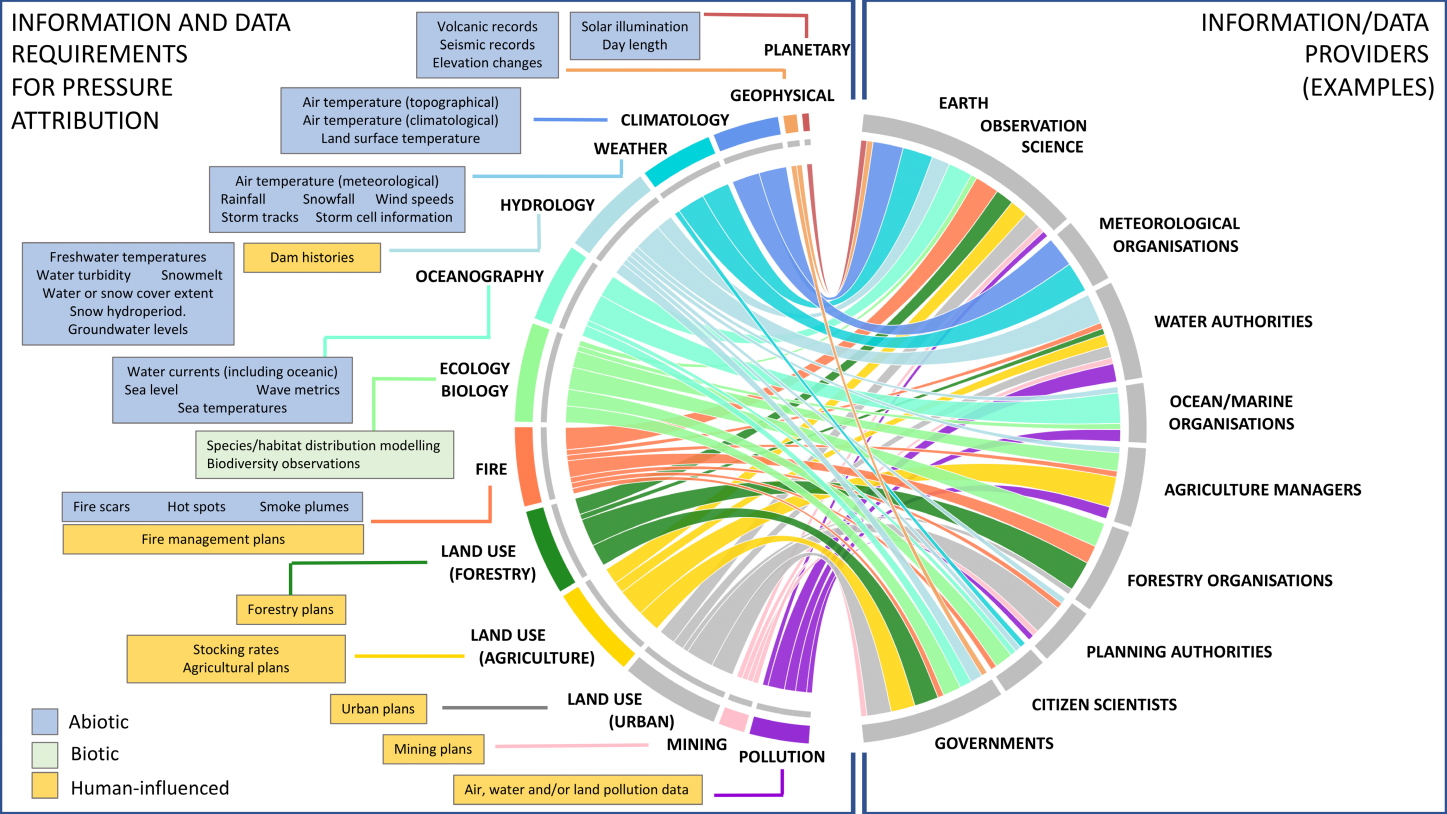
Chord diagram summarizing relationships between (i) primary information/data requirements (e.g., for climatology, hydrology, land use in domains of forestry, agriculture or urban) for attribution of pressures and (ii) information/data providers (right). Types of pressure data are grouped as primarily abiotic, biotic or human‐influenced.

Evidence for pressures was obtained primarily from AEDs (e.g., climate records) although reference was made to some EEDs, including summaries of these (e.g., hydroperiod maps) over and between different periods (e.g., to determine multi‐annual including decadal trends; Figure 5). Evidence for abiotic pressures was sourced primarily from the domains of meteorology, climatology, hydrology, oceanography, volcanology, seismology or fire ecology. These included long‐term climate records (e.g., trends or anomalies in temperature or snow fall), shorter‐term weather data (wind speeds, rainfall amounts) or marine data (e.g., sea‐level records, ocean acidity). Evidence requirements for biotic pressures related primarily to the distribution, abundance and ecological and behavioural characteristics of flora (e.g., invasive or native species) and fauna (including pathogens, herbivorous insects or vertebrates). Biodiversity or other observational records were also an additional source. Anthropogenic pressures often have the same or similar drivers (primarily economy). However, when compared with natural events and process, anthropogenic activities were found to be more diverse, disruptive, unpredictable and discrete (i.e., in that extent changes are commonplace) and also diverged from natural pathways and sequences. In this case, evidence requirements were more demanding and included, for example, management plans (e.g., in forestry, agriculture, mining, water, and urban settings), field observations and surveys (e.g., of biodiversity, plant health, invasive species) and measures of pollution (air, water and/or land, with examples being atmospheric nitrous oxide concentrations or presence of oil spills in coastal regions). The data used for determining pressures are diverse and usability depends on factors such as the area considered, the timing of change and relevance. However, indicative examples of data are the Essential Climate Variables available from the Global Climate Observing System [GCOS] (2022) and the European Space Agency (ESA) Climate Data Dashboard (ESA Climate Change Initiative [CCI], 2022), greenhouse gas concentrations (e.g., Ritchie & Roser, 2020), volcanic eruptions (e.g., Smithsonian Institution, 2022), global surface water metrics (Pekel et al., 2016), active fires (e.g., NASA Earth Observations, 2022), global‐scale mining polygons (Maus et al., 2020) and citizen science observations (e.g., Medlyn et al., 2019).

In all cases, spatial measures of change over longer periods of time (including those from remote sensing instruments) could be used as additional evidence for pressures. These included the past frequency of fires of different severity, water inundation or snow fall over different time periods (abiotic), vegetation age (as an indicator of growth or succession; biotic), or histories of deforestation, urban expansion or agricultural land use (anthropogenic; Prates‐Clarke et al., 2009). To obtain the evidence, often time‐series comparisons of EDs (e.g., the frequency/duration of vegetation lifeform presence, water or burn scars, climatic variables) were considered necessary to summarize change with outputs including periods of active land use, fire history, annual water hydroperiod and forest age. Many of these can be obtained from past and current ground and EO data. Locational and contextual information within the landscape was also highlighted as being important for the evidence base, particularly as many of the pressures arise from events, processes or activities that occur some distance away, spatially or in time.

In some situations, the sources of information for pressures overlapped with impacts, but their use remained distinct. For example, changes in annual precipitation amounts (the pressure) are often reflected in the impact of between‐year increases or decreases in annual water hydroperiod as a result of reduced or increased water flowing through a landscape. Changes in annual hydroperiods themselves can also be the pressure, leading to the impact of a reduction in vegetation amounts or extent.

#### 5.2.2 Evidence requirements

**FIGURE 6:**
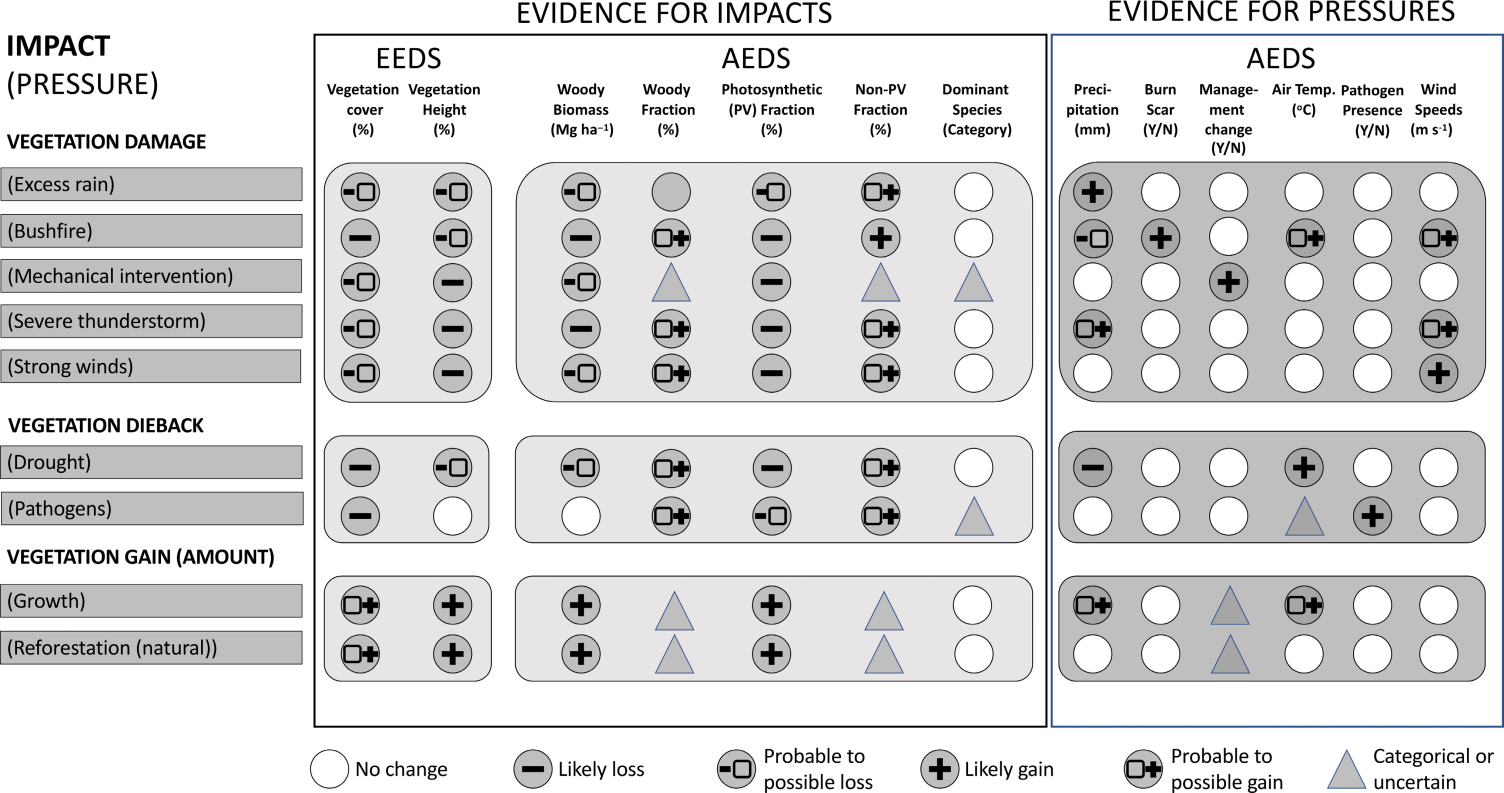
Diagrammatic representation of the accumulation of evidence for describing and assigning reasons for change, using vegetation damage, dieback and gain (amount) as examples. Similar evidence is accumulated for each of the ‘impact (pressure)’ categories, noting that the list of these as well as EDs is not exhaustive. EDs, Environmental Descriptors.

A diagrammatic representation of the way evidence can be accumulated to identify impacts and pressures is indicated in Figure 6. Generally, more than one ED (EEDs and AEDs) was required to provide the evidence for change, while others gave additional confirmatory evidence. While the evidence‐base indicated when and where an ED might be used to describe and define change, the selection of EDs as evidence was ultimately considered to be at the discretion of the user. These would often be dependent upon the knowledge and experience of those observing, the quality of records or measurements on the ground, the spatial resolution and/or temporal frequency provided by remote sensing instruments and their ability then to provide relevant EDs, and quantitative measures of robustness, including statistical assessments relating to uncertainty in retrieval or categorization (e.g., Comber et al., 2012).

The areas represented and definitions used in the Global Change Taxonomy were inherently scalable, as EDs with consistent units or categories were used in the evidence base. As illustration of this scale independence, dieback of leaves (whether as individuals, on a branch or a whole plant, or within a plot or region) can be inferred from the same accumulated changes in state descriptors (e.g., foliar chemistry, amounts of photosynthetic and non‐photosynthetic material, biomass), with these providing evidence of the impact. These are often measured with reference to benchmarks (typically amounts). Changes in plant species composition through succession may differ when summarized for a plot on the ground or from EO over a larger area, but the AEDs of dominant plant species or measures of species richness apply in each case.

#### 5.2.3 Factoring in time

Evidence for impacts often came from the time dimension. For example, flash floods, planting or harvesting of crops were, by definition, linked to shorter time periods while ecological succession and deglaciation were linked to processes that are ongoing and longer in duration. For this reason, summaries of EDs over defined periods (e.g., sub‐daily to centuries) were further found to be fundamental and important evidence for change, with examples, being seasonal/annual water or snow hydroperiod, maximum canopy cover, or average land or sea temperatures.

**FIGURE 7:**
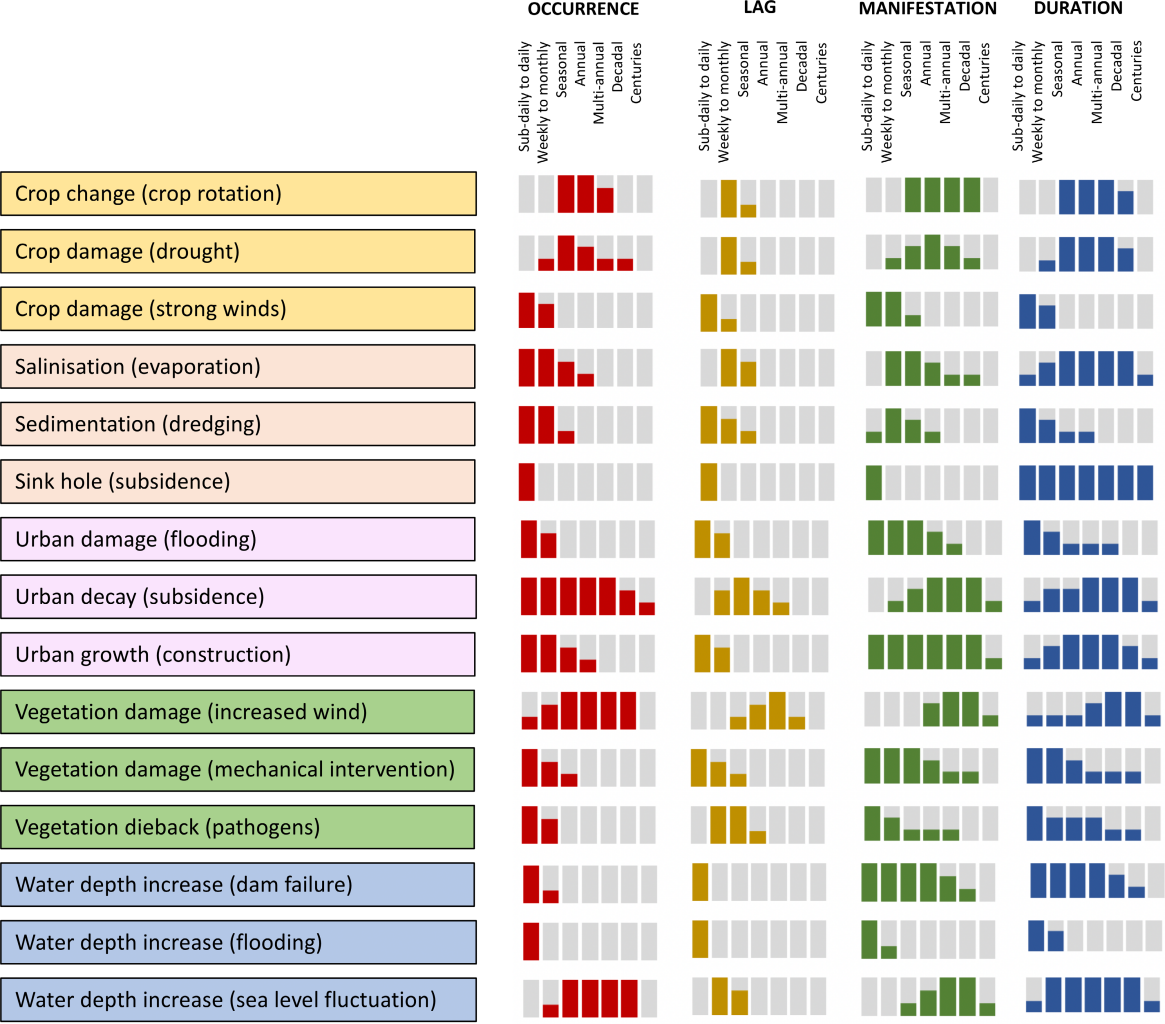
Examples of the varying time periods over which changes can be observed giving consideration to the occurrence, lag, manifestation and duration of the change. Changes are ranked by time interval (sub‐daily to daily, weeks to months [1–3], seasonal [>3 to 6 months], annual, multi‐annual, decadal and centuries). The histograms indicate, in relative and indicative terms, a high, medium or low likelihood of a natural event or process or human activity occurring in the specified time periods.

In developing the lines of evidence, descriptors of time (occurrence, lag, manifestation and duration) were found to vary and became important additional discriminators of impacts and pressures and valuable additions to the evidence set (Figure 7). Furthermore, by factoring in time, greater recognition was given to the temporal variability of landscape dynamics and how this crucially influences perceptions of change, including directions.

Many definitions of pressures were linked to the time of their occurrence. For example, human activities such as ploughing, tillage and planting (leading to impacts of bare soil exposure or crop establishment respectively) were typically associated with shorter periods of time, while changes in the extent of surface water could be indicative of a flash flood (hours), flooding (overflowing water, days to weeks), inundation (submerging; seasonal or annual) or sea level fluctuation (multi‐annual or decades). Several impact terms also reflected or inferred the timings of the occurrence of change, with examples being net snow gain or loss (extents) or damage to crops, both of which are typically confined to a season (e.g., the winter or growing period respectively). However, many did not reflect or reference the timings of events or processes (e.g., vegetation dieback).

Terms associated with a lag between the time of occurrence of an event, process or activity and its manifestation, whether on the ground or remotely, were often inherently associated with specific combinations of impacts and pressures. For example, coral bleaching, algal blooms or blackwater events, which occur in different environments (saltwater to freshwater), are often only recognized as becoming evident following the pressure of extended periods of high‐water temperature. Vegetation dieback resulting from pathogens is frequently assumed to only become evident several months or even years after the initial infestation (Jurskis, 2005).

The time over which change is manifested could also be inferred from several pressure terms. For example, thinning of trees is aimed at encouraging forest growth in the longer term but is inherently associated with a short‐term decrease in canopy cover that is often only evident (e.g., in EO data) for a few years until the canopy reforms (Olsson, 1994). However, the majority of changes where manifestation was time‐limited were linked to impact terms. For example, those conveying shorter periods of manifestation included snow accumulation (sub‐daily to weeks), algal bloom (typically weeks) and flooding (days to months), all of which are generally temporary in nature and their time of observability often coincides with the commencement and cessation of the event, process or activity. By contrast, the construction of roads or mines or buildings (reflecting urban sprawl) would be observed at the time of their construction, but the impact can be visible for many years, decades or even centuries after. Some changes only become evident after many years of observation, with deglaciation or impacts resulting from long‐term changes in climate (e.g., drought) being notable examples.

The duration of natural events and processes and human activities that lead to change integrates the periods of occurrence, lag and manifestation but could go beyond these. For example, while increases in vegetation amount associated with the positive pressure of regrowth might not be evident within remote sensing data in the later succession stages, the process could be perceived on the ground for decades after. In many cases, the duration of an event, process or activity ends abruptly. This was reflected in terms such as vegetation loss (extent) through deforestation or crop change in cultivated lands through ploughing. In others, longer‐term transitions are more common, particularly when natural long‐term processes occur. An example is deglaciation, which often transitions into a period of glaciation following changes in climate.

## 6 DISCUSSION

### 6.1 Requirements for a globally relevant change taxonomy

Regardless of scale, landscape change is often described at broad levels and within specific domains (e.g., forestry, hydrology and agriculture) or in relation to particular natural events (e.g., storms, floods) or processes (drought) or human activities (deforestation, urban expansion). While a number of the impact or pressure terms listed in the Global Change Taxonomy are often commonly referred to (either singularly or in combination) within these domains, they are usually only comparable at the broadest level, between locations and over time. This is largely because of a lack of consistency and standardization in terms used and variable provision and use of definitions (Nedd et al., 2021). Collectively, these compromise or limit dialogues on change across multiple communities. As illustration, since 1945, the Web of Science lists over 40,000 records that make reference to the topic land cover change (Table S3), with these encompassing primarily environmental sciences and studies (23.7%), geosciences (multi‐disciplinary) and geography (18.9%), and ecology, forestry and biodiversity conservation (14.8%). Other fields include water resources, meteorology/atmospheric sciences, agriculture and agronomy, soil science, urban studies and marine and freshwater biology. The majority of these studies are subject specific with a narrow focus that insufficiently convey or provide a generic capacity for describing change. Interestingly, 15.4% reference remote sensing or other imaging technologies, which acknowledges their increasing role in detecting, quantifying and describing land cover change over areas of varying size (often local to national/regional but also global) and different time frames.

The Global Change Taxonomy addresses some of these shortcomings by providing a consistent and easily understood framework that references and defines specific terms describing change. Defining the impacts and pressures separately also allows their use in new combinations depending on context. The provision of primary definitions for impacts and pressures within an online glossary ensures that these can be used consistently, appropriately and transparently. Term definitions can be modified or alternatives suggested based on new opinions, information or knowledge (from experts and non‐experts). Nevertheless, in recognizing that multiple definitions for the different impact and pressure terms may exist, with some being specific to countries or applications (as examples), these can be used as a replacement if required. Secondary definitions for terms and sources might be appropriate for some categories.

The Global Change Taxonomy has not been established as a replacement for already well‐established generic or domain‐specific frameworks, umbrellas and methods for describing and quantifying change, many of which are which are relevant and common to political, social, economic and environmental agendas. Instead, it intends to provide more detailed and consistent descriptions and information that support the interpretation and assessment of diverse changes occurring within landscapes and why these occur, in tandem enhancing capacity to improve comparison and aggregation. This is anticipated to stimulate the development of mechanisms for systematically and consistently attributing cause‐effect reasoning around impacts, flagging potential areas for concern, prompting management and policy considerations and better informing decisions.

The Global Change Taxonomy is also well placed to provide more detailed and comprehensive descriptions of higher‐level change terms, with land degradation and desertification being notable examples. Land degradation is defined by the Intergovernmental Science‐Policy Platform on Biodiversity and Ecosystem Services (IPBES) as the many processes that drive the decline of biodiversity, ecosystem functions or ecosystem services and its halt and reversal is one of the primary goals of the UN 2030 Agenda for Sustainable Development (Metternicht et al., 2020; Mosca et al., 2020; United Nations, 2015; SDG15). However, other definitions of land degradation have been put forward that highlight, for example, reductions in land or soil productivity as a result of human actions (Lal, 2009). Therefore, as a term, degradation has many connotations and interpretations, which typically depend on use cases and/or domain settings (Gibbs & Salmon, 2015) and encompasses a range of diverse and often complex processes (e.g., habitat condition assessment for terrestrial biodiversity; Harwood et al., 2016). Furthermore, definitions of degradation are directed towards specific environments, such as forests or wetlands, where reference is generally made to constituents of these. As examples, degradation of forests has been described as the persistent loss of carbon stocks (biomass) over time, with reference often made to the ED canopy cover to differentiate this process from deforestation where a change from forest to non‐forest occurs (GOFC‐GOLD, 2013; IPCC, 2003). For wetlands, definitions of degradation often refer to a temporary loss in extents and/or extensive amounts of water and/or intensive reductions in water quality. The attribution of pressures leading to degradation is also variable. For forests, these include selective timber harvesting of native forests, fuelwood collection, grazing within a forest, and fire (Pearson et al., 2017) while for wetlands, agricultural expansion and drainage and urban development are frequently cited (Bassi et al., 2014). These same issues are pertinent to other land covers, noting that over 75% of the terrestrial environment has been modified through human activities, either by loss or through degradation of the original ecosystems (IPBES, 2018), with every biome affected and most changes taking place over the past century (Willcock et al., 2016). Furthermore, desertification is associated with arid, semi‐arid and dry sub‐humid environments and is defined by the United Nations Convention to Combat Desertification (UNCCD, 1994) as a form of land degradation. However, desertification is again an umbrella term that considers a number of pressures arising from both climate and economic drivers, including deforestation, overgrazing, irrigation, reduced rainfall and increased temperatures with resulting impacts including losses in vegetation extent or amount, bare soil exposure, increased soil salinity and soil erosion. Desertification can also be temporary with reversals associated with an increase in vegetation extent and amounts. Indeed, Thomas (1997) highlighted that the use of the term desertification should either be discontinued or more precisely defined given its numerous definitions and application to a wide range of situations. These conflicts of definitions are addressed by the Global Change Taxonomy and specifically the use of impact and pressure combinations which allows for contributory natural events and processes and human activities to be described, singularly or in combination, and amalgamated to better describe desertification and more generally, the processes leading to degradation. These two examples (degradation and desertification) highlight that differences in definitions and descriptions and the lack of detail in many reduces collective understanding of how past and current land use practices and climate have led to loss and damage to ecosystems and environments. Our ability to construct pathways towards better management and use in the future is also compromised.

### 6.2 Scalability and evidence for change

The Global Change Taxonomy was developed so that it could be applied across multiple spatial scales (local to global), temporal frequencies (e.g., sub‐daily to decadal) and timeframes (past, present and future) and also connect evidence collected from measurements or observations on the ground with those acquired by airborne and spaceborne sensors. This was accomplished because both impacts and pressures are determined from differences in states, including those that relate to and define the pressures (e.g., temperature, sea level). In all cases, these are quantified using continuous and categorical EDs (i.e., states) with pre‐defined units or codes respectively that are scalable in space and time. Notable examples of these are percent (%, originating from the Romans), metres (m; defined in 1773) and the standard temperature scales of degrees Fahrenheit (°F; after Daniel Gabriel Fahrenheit in 1714) and Celsius (°C; Anders Celsius in 1742). These have been used for centuries and will continue to be recognized for the foreseeable future. The use of unit measures or codes within land cover and change classification systems is therefore essential for ensuring long‐term consistency and application. The inclusion of time in the evidence‐base is also particularly useful as it allows differentiation of specific categories listed in the Global Change Taxonomy. Noted here is that impacts relate more to the manifestation of change in situ while pressures consider influences that might also originate from a different location or area or be historically connected (in time).

As both continuous and categorical EDs provide the evidence for impacts (based on state changes) and pressures, the Global Change Taxonomy is fully applicable from point locations to entire areas. This capacity to consistently use the terms and definitions of the taxonomy across scales is highly relevant to decision making. It is noteworthy that some of the terms used in the definition of change might become redundant at particular scales or become more or less relevant as a function of the size, dimensions and geometrical arrangement of land units. For example, crop loss (due to farmland abandonment) or gain (through planting) is generally specific to the entirety of one or more fields. Vegetation loss through deforestation is only relevant to the surface area that previously supported forests. While impacts and pressures could largely be segregated, several classes belonged to both although the same definitions were used in each case. As an example, flooding can describe both an impact and a pressure.

As with other domains (e.g., socioeconomics and health), there is a requirement for environmental decisions and policies to be based on or informed by evidence. Hence, the establishment of the Evidence‐Based Change Framework to support use of the Global Change Taxonomy represents an important step forward in this direction. The combination of the taxonomy and associated glossary with terms that can be described from state changes and ascribed with different driving pressures provides a mechanism for identifying, selecting and combining evidence from a diverse range of sources. As such, the design and coherent structure provide unique capacity for consistent and robust use across multiple domains. As indicated, ground measured, remotely sensed or modelled EDs should only be accepted into the evidence‐base if they have pre‐defined units or codes respectively, are domain relevant and are validated (ideally with estimates of uncertainty). Appropriate identification and/or assignment of labels (i.e., positive, negative) to changes in continuous units, categorizations of these or just categories (i.e., where only a change can be registered) often depends upon the perceptions, experiences and opinions of observers at the ground level or the robustness or sensitivity of, for example, inventory methods, measuring devices or observing sensors. In this context, the directional labels assigned to EDs as they change over time need to be considered when using or advancing the evidence‐base for the different ‘impact (pressure)’ categories.

### 6.3 Progressions and dependencies of changes in states

**FIGURE 8:**
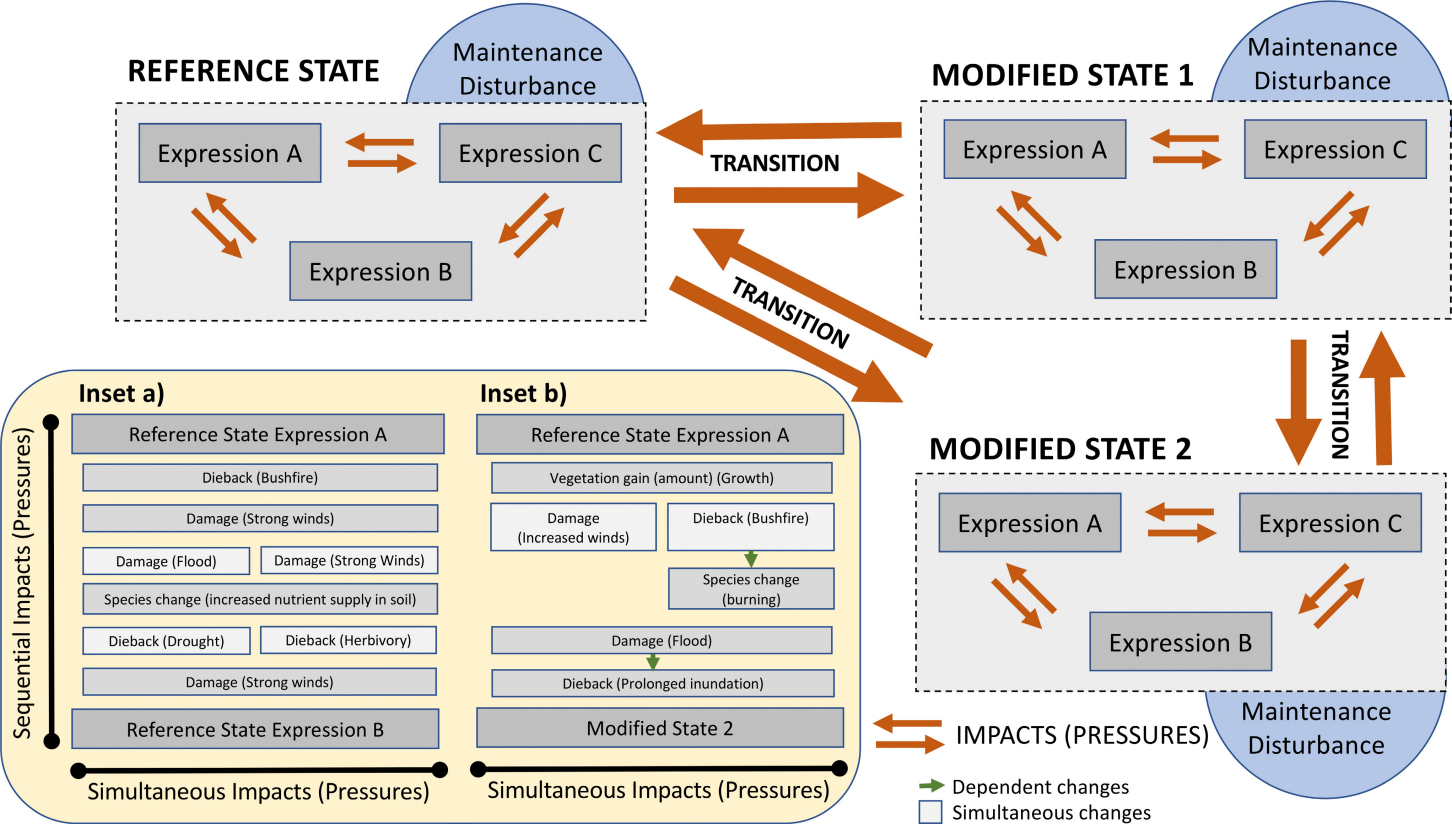
Template for defining state and transition models (adapted from Richards et al., 2020). Ecosystems within reference states may, at any point in time, be represented by different expressions (e.g., A–C) that are maintained by endogenous disturbance regimes. External pressures (exogenous disturbances) may cause the reference ecosystem to transition into a modified state, wherein different expressions represent transient variability. Examples of sequential and simultaneous changes (dependent and independent) that can lead to a transition between ecosystem reference state expressions (Inset a) and from a reference to a modified state (Inset b) are also provided.

The Global Change Taxonomy was found to accommodate changes that are dependent or independent of others, are discrete and singular, or occur simultaneously or sequentially (Figure 8, Insets a,b) albeit over varying spatial scales and time frames. Accordingly, linking of the impact and pressure categories provides more information on change than when used alone, with the time component being relevant in many cases.

Dependent changes were determined as requiring a previous form of change or another history of condition. In this case, each change depends on what has occurred before. For example, the change ‘water depth decrease (dam removal)’ can occur over periods ranging from hours to several months and may lead subsequently to ‘vegetation gain (extent) (colonization)’ of the exposed substrate by pioneer aquatic or terrestrial species followed by ‘vegetation gain (amount) (growth)’. This latter change would not have occurred without the former taking place. Independent changes result from different and not causally related pressures and may also have different drivers. For example, ‘crop damage (excess precipitation)’ might be followed by ‘vegetation dieback (pathogens)’ or ‘bare soil exposure (ploughing)’ . In each case, there may be no direct dependency on the events, processes or activities that have occurred beforehand.

Changes may also be discrete, singular and well defined e.g., ‘vegetation loss (urban construction)’, while others might be more complex, taking place either simultaneously or sequentially. For example, ‘vegetation gain (amount) (growth)’ can take place alongside ‘vegetation health improvement (irrigation)’; ‘vegetation loss (extent) (vegetation clearance)’ can simultaneously lead to ‘cropland gain (farmland creation)’. Sequential changes might be ‘water gain (extent) (flooding) followed by vegetation species change (prolonged inundation)’. Both dependent and independent changes, whether single or multiple (simultaneous or sequential), can occur over varying time frames, with time often providing a key contribution to evidence.

### 6.4 Simple versus complex pressures and impacts

The Global Change Taxonomy provided a standardized approach to describing both simple and complex changes. Simple changes were associated mainly with extent (between‐class) changes that are discrete, often expansive and frequently sequential in nature. Examples are “vegetation extent (loss) (deforestation)”, where vegetation is typically fully removed and then replaced by urban buildings, infrastructure and/or bare substrate surfaces, and “vegetation species change (crop rotation)”. In both cases, the OED can remain the same (e.g., natural or cultivated terrestrial or aquatic vegetation) but the lifeform EED (woody, herbaceous) can change. The affected area may undergo multiple changes in unison as the landscape evolves (e.g., within field units or forest coupes). Complex changes are considered to be more common to many natural ecosystems and more frequently associated with within‐class changes. As such, reference to a diverse range of EDs would be expected when gathering evidence for change.

One approach to systematically capture more complex changes is to align use of the Global Change Taxonomy with conceptual dynamic ecosystem models (commonly referred to as State and Transition Models; Westoby et al., 1989). As illustration, the Australian Ecosystem Models (AusEcoModels) Framework (Richards et al., 2020) defines and describes what are called dynamic reference states of ecosystems. Within these, changes resulting from endogenous disturbances (to which ecosystems have adapted and integrity is not disrupted) are captured as a set of different expressions (Figure 8), and each can be classified and described using EDs. Changes within or between expressions (simultaneous or sequential, dependent or independent) can also be described using the Global Change Taxonomy and associated Evidence‐Based Change Framework.

An example is the reference state of mangroves ecosystems, which exist in multiple expressions (Ward et al., 2016) across the tropics, subtropics and some temperate regions as a function of biogeography (Ellison et al., 1999; Yessoufou & Stoffberg, 2016), tidal regimes (Worthington et al., 2020) and climate (e.g., Simard et al., 2018). Within each mangrove reference ecosystem, shifts in expression (e.g., from A to B of the reference state in Figure 8) may occur because of sea level fluctuations, floods, droughts or storm events. The impacts of these (e.g., dieback, damage, vegetation gains or losses in extent) can be defined and/or quantified by comparing EDs over time (e.g., canopy cover [%] and height [m], non‐photosynthetic plant material [%] and dominant species). These and others describe the ecological composition, structure and function at different points in time, with their magnitudes or types often differing as a function of the stage of the disturbance cycle. Colonization or recovery following fluctuations in sea level (Duke et al., 2017) or cyclones (Asbridge et al., 2018) are notable examples. As these pressures occur over an extent, and at a frequency and intensity to which the ecosystems (in this case, mangroves) have adapted, the overall integrity of the ecosystem is maintained in each particular reference state.

Across the range of ecosystems and expressions, events and processes are dispersed in space and time and their diversity is generally maintained. However, if these events are widespread at the same time, ecosystem homogenization may occur, which often leads to degradation and shifts between expressions. These can also be driven by endogenous disturbance regimes and recovery processes (e.g., climate) but also by anthropogenic disturbances (e.g., Indigenous fire management) that lead to transformative changes in ecosystem structure and composition and transitions from reference states (and their different expressions) to dynamic modified states. These are further distinguished within the AusEcoModels framework (Richards et al., 2020). Such transitions can, in some cases, lead to changes between OEDs (conversions) as well as within (modifications). The collective characteristics of dynamic ecosystem reference state expressions provide benchmarks for quantifying departures that result from transitions within these and to modified states. The overall departure from these can be used as an index of quality or condition for a given purpose, such as assessing habitat condition (e.g., Harwood et al., 2016), and thereby inform the level of concern and resulting policy or management response. The Global Change Taxonomy can further be used to articulate changes that may lead to transitions with similar, different or collective outcomes over equivalent or differing timeframes. For example, several dimensional components of a forest (whether 1 ha or many km^2^ in area) may experience vegetation dieback from pathogens, herbivory, fire and/or drought while others may be experiencing vegetation growth through succession or the introduction of exotic or invasive species. By considering the different impacts and pressures in combination, the net effect can be better quantified, with this contributing to improved understanding of ecosystem dynamics (e.g., losses and/or gains in biodiversity and carbon). Such knowledge can also support modelling of vegetation dynamics, species distributions, processes and states and transitions.

### 6.5 Relevance to existing frameworks of environmental governance

The transition matrix used within the change framework and taxonomy is similar to that used by the Intergovernmental Panel on Climate Change (IPCC; Metternicht et al., 2020) for estimating and reporting greenhouse gas emissions and removals from the Agriculture, Forestry and Other Land Use (AFOLU) sector (IPCC, 2006). The AFOLU matrix considers six categories: croplands, wetlands, settlements and other lands along with forestlands and grasslands. The latter two are distinguished by the lifeform EED of the LCCS, which is not differentiated in Level 3 of the LCCS but included in the modular phase (i.e., Level 4) and as a type category. This EED can instead be included alongside the Level 3 classes in the base transition matrix so as to align with the IPCC reporting, thereby introducing an important descriptor and discriminator of change (e.g., woody to herbaceous or vice versa). Other EEDs could be equally added into the base transition matrix (e.g., transitions in water physical state, namely water, ice or snow). However, these are arguably best integrated (as is lifeform) within the later stage where evidence for change is gathered. In the IPCC, wetlands encompass both natural aquatic vegetation and water as well as cultivated or artificial counterparts respectively, which are distinguished and can be further described using the FAO LCCS.

The Global Change Taxonomy and Evidence‐Based Change Framework provides more detail compared with many preceding methods and other taxonomies for describing change and can therefore inform agendas that aim to reduce or reverse adverse impacts on the planetary environment or mitigate/adapt to these. These refer to ameliorating the impacts of human activities (e.g., on ecosystem integrity and biodiversity) but increasingly consider the accelerating effects of climate change. Amongst these are the UN Sustainable Development Goals, which focus on addressing land degradation, and the Ramsar Convention, which seeks to ensure wise use of the World's wetlands.

### 6.6 Application to EO

The combined use of impacts and pressures to describe change was developed initially as a theoretical framework but then extended so that ground‐based observations and interpretations could be scaled, including through EO even though timeframes of observation become more rigid. The use of EO‐derived EDs for constructing land cover classifications according to the FAO LCCS was suggested by Lucas and Mitchell (2017), who demonstrated its implementation for protected areas in Europe under the auspices of the Earth Observation Data for Ecosystem Monitoring (EODESM). Developed originally through the European Union's BIO_SOS (Lucas et al., 2014) and its successor, Ecopotential, EODESM provided a mechanism by which the EEDs of the LCCS (e.g., plant lifeform, cover, height, leaf type, water hydroperiod), when obtained separately, could be combined subsequently to generate detailed spatial characterizations of landscapes across multiple spatial scales and for multiple points in time.

Unlike previous classification approaches, EODESM was uniquely able to access and use the many thousands of categories defined by the FAO LCCS (Owers et al., 2021), each of which has a biophysical meaning to generate comprehensive classifications of land cover. This capacity was further demonstrated by Lucas et al. (2019), who initially conveyed the principles behind EODESM and applied the approach to map land covers for selected sites in Australia by integrating EDs retrieved or classified from dense time‐series of Landsat sensor data. Owers et al. (2021) subsequently showed how EODESM could be applied continentally across Australia using Landsat sensor data acquired in 2010 and 2015, which then paved the way for full continental mapping annually from the entire Landsat archive held within Digital Earth Australia (DEA) and using Open Data Cube (ODC) functionality (https://www.opendatacube.org). This series of national maps of land cover were produced annually for 34 years (1988–2020) and released openly and publicly in March 2022 (https://www.dea.ga.gov.au/products/dea‐land‐cover). Implementation of EODESM was undertaken in parallel with Living Wales (wales.livingearth.online), with DEA and Wales (UK) using the same software, and Wales establishing a dedicated ODC of Landsat, Sentinel‐1 radar and Sentinel‐2 optical data. A particular advantage of developing EODESM through the ODC is that this geospatial and data management and analysis software and architecture is open source and has been developed for application in many countries (e.g., Switzerland, Cambodia, Vietnam) and regions including Africa (through Digital Earth Africa) and the Pacific Islands (Digital Earth Pacific). Therefore, integration within the ODC provides the capacity to implement this elaborate taxonomy and framework across areas of varying size (local to continental) and over different spatial and temporal scales.

Given that EODESM can generate scalable land cover classifications from continuous or categorical EDs with defined units or codes, this system is able to use the Evidence‐Based Change Framework to detect and differentiate the impact and pressure categories listed in the Global Change Taxonomy, either singularly or in combination, from time‐series of EO and other spatially explicit layers. Initial demonstrations of capacity and future potential included the detection and description of mangrove dieback in northern Australia's Gulf of Carpentaria and reductions in reservoir water levels near Townsville, Queensland (Lucas et al., 2019), detection of clear‐cutting and flooding in Wales (Planque et al., 2020), and tracking mangrove dynamics in commercial rotational forests in Malaysia (Lucas et al., 2020). The proposed change attribution framework covers all land and water types and provides an accompanying interpretation of change observed in EO datasets, with this fulfilling a gap in the practical application and use of EO time series and facilitating consistency in future data collection efforts.

The change framework has also been developed to contribute information on land cover and use that can be used to interpret trends and is well positioned to support existing and new time series analyses. In particular, the framework can improve (i) summaries of change (e.g., in fire frequency, periods of active land use, water and snow hydroperiod, forest age) and (ii) assessment of trends, either in EO data or derived metrics/EDs. The framework can also be used in conjunction with techniques (e.g., the Breaks for Additive Season and Trend [BFAST]; Verbesselt et al., 2012) that identify break points in time‐series of EO data as indicators of disturbances (whether from natural events or human activities), track natural processes taking place over long time‐periods (Giuliani et al., 2020; Trends.Earth, 2018) or retrieve trajectories from land use and land cover maps generated using data of different modalities and according to a range of taxonomies (e.g., Zioti et al., 2022). Although established independently of the International Union for the Conservation of Nature (IUCN) and Conservation Measures Partnership (CMP) classification of direct threats and actions (Salafsky et al., 2008), the framework is highly complementary and further supports conservation actions (e.g., avoidance of negative impacts and pressures and promotion of those that are positive).

To support the validation of both land cover and change from EO data, the EarthTrack mobile application has been developed jointly by Wales and Australia with input from Ecopotential partners. EarthTrack was developed originally for recording global land cover but now incorporates the Global Change Taxonomy and the associated glossary. Inclusion within this mobile application thereby provides a mechanism for informing land cover and change classifications but also introduces the concepts, terms and definitions to a wide range of stakeholders. EarthTrack is being made available in 2022 and will be accessible through the primary app stores.

## 7 CONCLUSIONS

Through the integration of the DPSIR framework with structured land cover classification systems (e.g., the FAO LCCS), a new globally applicable taxonomy and associated framework for consistently describing land cover change across scales and time has been developed and is proposed for worldwide application. The Global Change Taxonomy, which is available online at 10.5281/zenodo.6884999, can support land cover change assessments on the ground level and from airborne and spaceborne observations, and also provides a mechanism for describing change based on predictions (e.g., from process models). The combined use of consistent impacts and pressure terms with clear and understandable definitions ensures that causes and consequences of change can be consistently described regardless of the environment being considered.

The Global Change Taxonomy currently lists 246 impact (pressure) combinations, and other terms or combinations can be added as needs arise and following peer review. For each combination, a comprehensive assessment of the evidence needs for discerning the impact and defining the pressure has been suggested. For each, different types and sources of information are typically needed, although there is overlap in some cases. The evidence for change can be progressively accumulated through comparison of OEDs, EEDs and AEDs between any two time‐separated periods, which allows changes in extent, extensive and/or intensive amounts and types of land covers and their components to be quantified. The integration of time within the evidence‐base allowed links with impact and/or pressure terms that inherently include a time dimension. The inclusion of time descriptors (occurrence, lag, manifestation and duration) also provides greater flexibility in describing and differentiating change impacts and pressures.

The Global Change Taxonomy and supportive Evidence‐Based Change Framework can be used across multiple domains, including policy, land management, conservation and restoration, and land use planning. The envisaged audience includes (i) policy and decision makers who are seeking to understand the reasons for change observed from remote sensing in a standardized framework and when to initiate actions based on concerns; and (ii) the public (including citizen scientists) who are anxious to collect in situ data that consistently support the development of land products from EO data and advancement of their use for addressing the major challenges of today (e.g., climate change, sustainable development, biodiversity loss). Furthermore, as standardized data on change are collected over time, these can provide an attribution framework that can allow scientists and others with a basis for meta‐analysis that can be used to better understand and inform change processes and the cumulative impacts or benefits in different regions.

The use of common, easy to understand and standardized terms and definitions in the taxonomy and the inclusion of EDs with pre‐defined units or categories within the evidence base provides a unique opportunity for communities to generate, exchange and discuss land cover change at local to global scales and openly collaborate. Terms used in the conceptualization of the approach and relating to land cover classifications and descriptions and temporal changes associated with natural events or processes or human activities are also summarized in Tables S4 and S5. Collectively, these help to better define change processes, many of which encompass multiple impacts and pressures such as land degradation and desertification. Furthermore, capacity is introduced to plan and enact future changes and monitor progress towards goal achievement (e.g., in restoration, sustainable resource management), particularly as ground‐based and remote observations can be captured and coupled with modelled predictions or plans.

We hope that the Global Change Taxonomy and Evidence‐Based Change Framework will provide a foundation for harmonizing change assessment methods across sectors and help promote efficiency in remote sensing of land cover as part of integrated and collaborative efforts. In recognizing the substantive benefits of using the taxonomy and framework (e.g., in conservation, restoration, sustainable land management), our ongoing research and development will continue to focus on demonstrating practical application from user to regional scales, with this aimed at widening and encouraging future adoption.

## CONFLICT OF INTEREST

The author declares that there is no conflict of interest.

## Supporting information


Table S1
Table S2
Table S3
Table S4
Table S5

## Data Availability

The Global Change Taxonomy (terms and definitions) listed in this that support the findings of this study are available at https://doi.org/10.5281/zenodo.6884999.
