# Supplementary material for: A globally relevant change taxonomy and evidence‐based change framework for land monitoring
Source: Glob Chang Biol. 2022 Sep 1;28(21):6293–317. doi: 10.1111/gcb.16346 (PMC9805224; doi:10.1111/gcb.16346)
Supplement: Supplementary file 1 — Table S1 Table S2 Table S3 Table S4 Table S5 [file GCB-28-6293-s001.docx]

Table 1. Global Change Taxonomy: Pressure terms and definitions. The time component for each term is indicated in the evidence-base.

| **Term** | **Definition** |
| --- | --- |
| Abandonment of fertilizer application | Cessation of the use of a substance (such as manure or a chemical mixture) used to make soil more fertile. |
| Abstraction | Removal of water from a water store. |
| Afforestation | Establishment of forest through planting and/or deliberate seeding on land that, until then, was under a different land use. Implies a transformation of land use from non-forest to forest. |
| Agricultural expansion | Increase in area of arable lands and pasture used for food production. |
| Agricultural loss | Decrease in area of arable lands and pasture used for food production. |
| Amenity development | Development of land for human use, includes golf courses and sports field. |
| Anchoring | Securing a boat firmly to the seabed using an anchor. |
| Animal stock change | Stock change (introduction, type change or removal) |
| Aquaculture expansion | Increase in the area of farms used for rearing of fish, shellfish, and some aquatic plants to supplement the natural supply. |
| Aquaculture loss | Loss of areas farmed for aquatic organisms, including fish, molluscs, crustaceans and aquatic plants. |
| Atmospheric deposition | The process whereby precipitation (rain, snow, fog), particles, aerosols, and gases move from the atmosphere to the Earth's surface. |
| Burning | On fire. |
| Bushfire | Grass fires, forest fires and/or scrub fires started naturally (such as by lightning), accidentally, or deliberately (such as by arson), but not in accordance with planned fire management prescriptions. Also called wildfire or unplanned fire. |
| Bushfire recovery | Regrowth after a wildfire, whether natural or intentional (e.g., for conservation purposes) resulting in an increase in the biomass or health of vegetation. |
| Cold snap | Below-average temperature of the land surface, inland waters or oceans. |
| Colonisation | Occupation of a habitat or territory by a biological community or of an ecological niche by a single population of a species. |
| Construction | Creation of infrastructure such as railways, roads, mines, buildings, agricultural farms, aquaculture farms or dams; includes barrages and marina development. |
| Control of invasive or exotic species | Management aimed at reducing or eliminating terrestrial or aquatic invasive flora and fauna species through activities such as weeding, applying pesticides or trapping. |
| Coppicing | Area of closely planted trees that are cut back regularly to provide wood. |
| Crop rotation | Alternating between types of crops on a seasonal basis in the same area to limit soil degradation and to help control weeds, diseases and pests. |
| Dam failure | The uncontrolled release of a water storage. The failure may consist of the collapse of the dam or some part of it, or excessive seepage or discharges. |
| Dam removal | Demolition/decommissioning of a dam enabling reinstatement of unconstrained water flow and typically reducing pooled water. |
| Decreased acidity | An increase in the pH of water or soils across the acidic range of 0 to 7. Inversely related to alkalinity. |
| Decreased alkalinity | A reduction in the pH of water or soils across the alkaline range of 7-14. Reduced buffering capacity of a water body; a measure of the ability of the water body to neutralize acids and bases and thus maintain a fairly stable pH level. |
| Decreased nutrient supply in soil | Reduced availability of nutrients needed for plants to grow or maintain physiological function and potentially influencing vegetation composition. Soil is a major source of nutrients needed by plants for growth. The three main nutrients are nitrogen (N), phosphorus (P) and potassium (K). |
| Decreased precipitation | Decreased condensation of water vapour in the atmosphere forming rain, snow, sleet or dew. |
| Decreased temperature | Decreased amount of heat in a place. |
| Deforestation | The permanent clearance of a forest, usually rapidly by cutting or burning over a large area, without replanting or natural regeneration. Also known as forest clearance. |
| Deposition | Deposition is the laying down of sediment carried by wind, water, or ice. Sediment can be transported as pebbles, sand & mud, or as salts dissolved in water. Salts may later be deposited by organic activity (e.g. as sea-shells) or by evaporation. |
| Dredging | Removing silt and other material from the bottom of bodies of water |
| Drought | Areas considered to be suffering from a serious or severe rainfall deficiency. |
| Drying | Prolonged shortage of water that dries out water-dependent environments. |
| Earthquake | The vibrations of the Earth caused by the passage of seismic waves radiating from some source of elastic energy. |
| Ecological restoration | The process of assisting the recovery of an ecosystem that has been degraded, damaged or destroyed including through mechanical habitat rejuvenation. |
| Encroachment | Increase in woody plant density or extent so that the natural equilibrium of woody plant layer (trees and shrubs) and herbaceous (grass and forb) layer densities is shifted towards woody species. |
| Erosion | Geological process in which earth materials are worn away and transported by natural forces such as wind or water. |
| Eutrophication | A natural process that results from accumulation of nutrients in lakes or other bodies of water. |
| Evaporation | Water changing from a liquid to a gas. |
| Excess precipitation | Above-normal increase in the amount of rain or snowfall. |
| Excess rain | Heavy rainfall associated with the passage of a storm or tropical cyclone than can produce extensive flooding. |
| Fallowing | A farming technique whereby a field is left unoccupied by crops during an entire growing period or part of it to raise soil fertility, accumulate moisture in the soil, increase the yield of all crops in a rotation and improve the quality of agricultural products. |
| Farmland abandonment | The cessation of agricultural activities on a given surface of land. |
| Farmland creation | Conversion of land to commercial, subsistence, intensive or extensive farming. |
| Fertilizer application | Enrichment of soils to increase productivity or health of vegetation (e.g., through slurry spreading). |
| Flooding | The inundation of water over land that is generally dry. |
| Fracking | Fracking is the process of drilling down into the earth before a high-pressure water mixture is directed at the rock to release the gas inside. |
| Frost | Deposit of soft white ice crystals or frozen dew drops on objects near the ground; formed when surface temperature falls below freezing point. |
| Fuelwood collection | Extraction of wood for fuel by collecting dead wood or by harvesting trees or their branches. |
| Grazing (natural) | Repetitive consumption of herbaceous or woody vegetation by wild herbivores/omnivores. |
| Grazing (stock) | Repetitive consumption of herbaceous or woody vegetation by domesticated herbivores/omnivores. |
| Greenspace construction | Replacement of urban built spaces with vegetation. |
| Ground water extraction | The process, deliberate or inadvertent, of extracting ground water from a source at a rate so in excess of the replenishment that the ground water level declines persistently, threatening exhaustion of the supply or at least a decline of pumping levels to uneconomic depths. |
| Ground water recharge | The process by which external water is added to the zone of saturation of an aquifer, either directly into a formation or indirectly by way of another formation. |
| Growth | Increases in cell size and number that take place during the life history of an organism. |
| Gypsum application | Spreading of a naturally occurring mineral that contains calcium and sulphur, thereby increasing porosity of the soil. |
| Harvesting | Act of removing a crop from where it was growing and moving it to a more secure location for processing, consumption, or storage. |
| Heatwave | A period of abnormally and uncomfortably hot and unusually humid weather. Typically, a heat wave lasts two or more days. |
| High inland water temperatures | Water temperatures of rivers, lakes, floodplains, reservoirs, wetlands, and inland saline systems that are warmer than usual, often based on the recent average. |
| Idle or fallow in rotation | A crop sequence that includes idle, diverted, or fallowed land in 1 or more of the previous years. |
| Increased acidity | A decrease in the pH of water or soils across the acidic range of 0 to 7. Inversely related to alkalinity. |
| Increased alkalinity | An increase in the pH of water or soils across the alkaline range of 7-14. Increased buffering capacity of a water body; a measure of the ability of the water body to neutralize acids and bases and thus maintain a fairly stable pH level. |
| Increased investment | Purchase of an asset, item or service. |
| Increased nutrient supply in soil | Increased availability of soil nutrients needed for plants to grow or maintain physiological function and potentially influencing vegetation composition. |
| Increased precipitation | Increased condensation of water vapour in the atmosphere forming rain, snow, sleet or dew. |
| Increased temperature | Increased amount of heat in a place. |
| Increased traffic | A greater number and/or frequency of vehicles moving over an area. |
| Increased wind | A higher velocity of air movement into an area associated with, for example, cyclones, hurricanes, typhoons, tornados, thunderstorms, storms or ice storms. |
| Insect herbivory | Increased presence of plant-consuming insects in an area. |
| Inundation | Progressive submergence of land or structures through flooding. |
| Inundation following extended drought | Progressive submergence of land or structures through flooding and following an extended period of drought. |
| Invasive or exotic species | A plant or animal that has been introduced into a region (terrestrial or aquatic) in which it does not naturally occur and that becomes established and spreads, displacing naturally occurring species. |
| Irrigation | Artificial supply of water to crops, horticulture or pastures. |
| Land reclamation | The restoration of productivity or use to lands that have been degraded by past human activities or have been impaired by natural phenomena. |
| Landslide | Movement of material downslope in a mass. |
| Levelling | Flattening ground by infilling or cutting the surface. Used to create a straight base for a new development including some forms of agriculture. |
| Mechanical intervention | Use of machines (e.g., to remove or alter vegetation) |
| Melting ice sheets/glaciers | Loss in the extent and volume of frozen water floating on or within sea water or accumulated on mountainous coastal or inland areas. |
| Mine abandonment | Land formally used for extractive industry but no longer in use and no new use observed. |
| Mine site rehabilitation | Design and construction of landforms as well as the establishment of sustainable ecosystems or alternative vegetation, depending upon desired post-operational land use. |
| Mining | Extraction of minerals, precious stones or coal, either through open cuts or deep shafts. |
| Mowing | Cutting down (grass, crops, etc) with a hand implement or machine |
| Natural diurnal and seasonal cycles | Natural processes or events that typically reoccur each day (diurnal) or within a year (seasonal). Examples are air temperature or solar illumination. |
| Non-insect herbivory (natural) | The process whereby an animal (non-insect) eats a plant or a plant-like organism such as a seaweed or phytoplankton. |
| Nutrification | The process by which water bodies, such as within an estuary or embayment, receive excess nutrients from a variety of sources (primarily agriculture, aquaculture and sewage), setting off a cascade of environmental changes. |
| Ocean-atmosphere oscillations | Naturally occurring cycles affecting sea surface temperature and precipitation |
| Overgrazing (natural) | Excessive grazing by natural animal populations without a sufficient break in time, resulting in declines in the biomass or health of vegetation. |
| Overgrazing (stock) | Excessive grazing by domesticated stock without sufficient rest, resulting in declines in the biomass or health of vegetation. |
| Pathogens | Any small organism, such as a virus or a bacterium that can cause disease. |
| Pesticide application | Use of a substance that is intended to kill, repel or otherwise control any organism that is designated a “pest”, including weeds, insects, snails, rodents, fungi and bacteria. Includes herbicides, which is a pesticide that is used to kill plants, or to inhibit their growth or development. Also known as weedkillers. |
| Planting | Placement of seeds, seedlings or saplings into the ground so they can grow, leading to an increase in vegetation amount or a change in plant species composition. |
| Ploughing | Making furrows in land mechanically or with an animal-drawn plough, by breaking and turning over the soil. |
| Pollution | Introduction of harmful materials known as pollutants into the environment. Pollutants can be natural, such as volcanic ash. They can also be created by human activity, such as trash or runoff from industrial sources (e.g., factories, farms, etc.) and contaminated with substances including fertilizers and pesticides/herbicides. Pollutants damage the quality of air, water, and land. |
| Prescribed burn | The process of planning and applying fire to a predetermined area, under specific environmental conditions, to achieve a desired outcome. |
| Prolonged inundation | Cumulative influx of water and increase in water depth for long periods, over an area that previously without water. |
| Prolonged snow cover | Cumulative gain of snow cover over extended periods. |
| Prolonged temperature decrease | Seasonal to multi-annual decreases in temperature compared with past averages. |
| Prolonged temperature increase | Seasonal to multi-annual increases in temperature compared with past averages. |
| Reduced investment | Sale of an asset or item or removing funding for a service. |
| Reduced or cessation of grazing | Reduction or cessation of repetitive consumption of herbaceous or woody vegetation by herbivores/omnivores. |
| Reduced runoff post flood | Reduced quantity of water discharged in surface streams following a flood event. Runoff includes not only the waters that travel over the land surface and through channels to reach a stream but also interflow, the water that infiltrates the soil surface and travels by means of gravity toward a stream channel (always above the main groundwater level) and eventually empties into the channel. Runoff also includes groundwater that is discharged into a stream; streamflow that is composed entirely of groundwater is termed base flow, or fair-weather runoff, and it occurs where a stream channel intersects the water table. |
| Reduced snowfall | Reduced precipitation falling as snow. |
| Reforestation (native) | The process of deliberate replanting of vegetation, often with a diverse species mix, on land where the previous plant communities have been cleared. Generally undertaken to improve environmental conditions. |
| Reforestation (plantations) | Establishing plantations of trees or shrubs (limited numbers of native or exotic species) for production or environmental and resource protection purposes. Includes farm forestry. |
| Regrowth | Natural increase in the number and size of plants over an area that previously supported vegetation that had been cleared or degraded. |
| Rehabilitation | The process of returning the land in a given area to some degree of its former state, after some process (industry, natural disasters etc.) has resulted in its damage |
| Removal of herbivores | The elimination of animals (insect and non-insect) feeding on living plant parts. |
| Repairing damage | Improvement in condition of infrastructure or an increase in amount of construction following damage repair. |
| Revegetation | The re-establishment of vegetation (excluding forests) in areas that have been cleared or highly modified. The mix of plant species may not be the same as that of the original vegetation. |
| Sea defence construction | Creation of infrastructure for the purpose of coastal protection. |
| Sea level fluctuation | Irregular rise and fall of sea level. |
| Sediment transport | The movement of solid particles (sediment) caused by movement of the water volume within which they are contained and the force of gravity. |
| Sedimentation | Deposition of sediment from flowing water (in channels or floodplains) or standing water (in wetlands, lakes, or oceans). |
| Selective logging | A type of (exploitation) cutting that removes only certain types of trees (a) above a certain size, (b) of high value, or (c) of specific size for specific purposes. |
| Severe thunderstorm | Severe thunderstorms are very localised events, not usually affecting wide areas as tropical cyclones and floods do, so their devastating impact is often underestimated. |
| Snow accumulation | Net increase in ice crystals or snow resulting from consecutive precipitation below or at near zero land and/or air temperatures. |
| Snowfall | Where water vapour turns into solid ice crystals and falls as snow. |
| Snowmelt | Water produced by the melting of snow. |
| Soil salinisation | The accumulation of or increase in the concentration of soluble salts (e.g., sodium chloride) in soil. |
| Storm surge | An abnormal rise in sea level over and above the normal (astronomical) tide levels. It can be thought of as the change in the water level due to the presence of a storm. These powerful ocean movements are caused by strong winds piling water up against the coast as a cyclone approaches. |
| Strong winds | A prolonged period of average wind speeds exceeding 40km/h during the day. |
| Stubble burn | An area with straw stubble that remains after grains (e.g., paddy, wheat) have been harvested and which has been intentionally set on fire. |
| Subsidence | The lowering of the Earth's surface, caused by factors such as compaction, a decrease in ground water or earthquakes. |
| Succession | The natural, sequential change (stage) of species composition in an ecosystem in a given area. |
| Thermal expansion | Increase in linear dimensions of a solid or in volume of a fluid because of rise in temperature. |
| Thinning | Removal or death of plants or parts of plants to decrease plant density in a stand and facilitate growth of remaining plants. Natural thinning can occur. Plant parts may not be removed from the area. |
| Tillage | Preparation of soil for agricultural use through mechanical intervention such as digging, stirring, and overturning. Tillage disrupts the soil structure in a way that increases porosity and accelerates soil erosion. |
| Topsoil removal | Taking away the surface layer of soil that contains partly decomposed organic debris, and which is usually high in nutrients, containing many seeds, and is rich in fungal mycorrhizae. Topsoil is usually a dark colour due to the ‘organic matter’ present. In arable land, ‘topsoil’ refers to the soil down to plough depth. |
| Tropical cyclone | A non-frontal low-pressure system of synoptic scale developing over warm waters having organised convection and a maximum mean wind speed of 34 knots or greater extending more than half-way around near the centre and persisting for at least six hours. |
| Undergrazing (by stock) | Grazing (by stock) at a level where there is evidence of the annual growth not being fully utilised or scrub or coarse vegetation is becoming evident and such changes are detrimental to the environmental interest of the site. |
| Undergrazing (natural) | Grazing (natural) at a level where there is evidence of the annual growth not being fully utilised or scrub or coarse vegetation is becoming evident and such changes are detrimental to the environmental interest of the site. |
| Urban fire | Urban fire occurs primarily in cities or towns with the potential to rapidly spread to adjoining structures. These fires damage and destroy homes, schools, commercial buildings, and vehicles. |
| Urban greening | Increase in the amount and extent of vegetation in urban settings within parks and urban farms resulting from the planting trees and other forms of vegetation. |
| Urban rehabilitation | Area devoted to regenerating and conserving the built heritage or the urban environment, including the ecosystems. In addition to the refurbishment of historical buildings and townscapes, such activities also comprise the modernisation and upgrading of technical facilities and the respect of environmental and security norms and standards. Includes urban restoration. |
| Variation in temperature | Fluctuations in temperature around a reference level. |
| Vegetation clearance | Removal of non-forest vegetation from an area to allow new land uses. |
| Vegetation thickening | Increasing shrub and tree density on grazed rangelands, woodlands and forests that may or may not have supported such woody plant populations in the past. |
| Volcanic eruption | The discharge (aerially explosive) of fragmentary ejecta, lava and gases from a volcanic vent. |
| Waste dumping | The disposal of solid wastes without environmental controls. |
| Wave action | Disturbance on the surface of a liquid body, as the sea or a lake, in the form of a moving ridge or swell that impacts on the structure of land elements. |
| Water movement change | A change in the direction of flow of a body of water that might be accompanied by a change in velocity, turbulence and/or discharge. |
| Water salinisation | The accumulation of or increase in the concentration of soluble salts (e.g., sodium chloride) in water bodies. |
| Weed invasion | Invasions begin when an introduced species is able to survive and give rise to subsequent generations. |
| Wetland drainage | Removal or exclusion of water from a wetland by pumping, excavation of channels, planting fast growing non-wetland trees or plants in a wetland area, abstraction of water from a river entering a wetland, channelling, reclamation and drainage itself. |
| Wetland restoration and/or construction | The process of assisting the recovery of wetlands that has been degraded, damaged or destroyed. |

Table 2. Global Change Taxonomy: Impact terms and definitions

| **Term** | **Definition** |
| --- | --- |
| Accretion | The build-up of sediments to form land or shoaling in coastal waters or waterways. It may be either natural or artificial. Natural accretion is the build-up of land on the beach, dunes, or in the water by natural processes, such as waves, current and wind. Artificial accretion is a similar build-up of land resulting from built structures such as groynes or breakwaters, or activities such as filling and beach nourishment, or also aggradation. |
| Algal bloom | A sudden proliferation of algae (microscopic plants) that occurs near the surface of a body of water. Blooms can occur due to natural nutrient cycles or can be in response to eutrophication or climate variations. |
| Algal dieback | Loss of algae (microscopic plants) that occurs near the surface of a body of water. |
| Bare soil exposure | Appearance of underlying soil following human or natural disturbance, such as removal of top soil prior to urban construction or grazing of natural vegetation. |
| Blackwater event | When organic material is inundated or washed into waterways and consumed by bacteria, leading to a sudden depletion of dissolved oxygen in the water. |
| Browning | A decrease in the amount or loss of photosynthetically active pigments (primarily chlorophylls) in vegetation. |
| Building or infrastructure abandonment | Buildings that are no longer habited or in use. |
| Compaction | Process that brings about an increase in soil density or unit weight, accompanied by a decrease in air volume. |
| Coral bleaching | When the coral host expels its zooxanthellae (marine algae living in symbiosis with the coral) in response to increased water temperatures, often resulting in the death of the coral. |
| Coral damage | Physical damage or loss of a coral reef that is not the result of changes in water temperature. |
| Coral recovery | Re-instatement of the previous extent or health of reef-building corals. |
| Crop change in cultivated lands | Replacement of one crop type by another. |
| Crop damage | Loss of or harm to crops during or following a disturbance. |
| Crop establishment | Planting of crops in an area on ploughed land. |
| Cropland gain | Increase of total cropping area or total area sown. |
| Cropland loss | Decrease of total cropping area or total area sown. |
| Decreased wave action | Decreased disturbance on the surface of a liquid body, as the sea or a lake, in the form of a moving ridge or swell. |
| Deglaciation | The uncovering of glaciated land because of melting or sublimation of a glacier. |
| Desalinisation | The process of soils becoming less salty; the reduction of soluble salts in water bodies. |
| Desertification | Reduction in the productivity of the land that is not reversible. In other words, land is desertified when it can no longer support the same plant growth it had in the past, and the change is permanent on a human time scale. |
| Elevation change | Change in the altitude above a reference sea or ground level. |
| Erosion | Geological process in which earthen materials are worn away and transported by natural forces such as wind or water. |
| Flooding | Overflow of water across an area. |
| Geomorphological change | A topographic adjustment that changes the geometry of landforms. Examples include estuarine evolution and beach rotation in coastal areas and mountain uplift. |
| Glaciation | Expansion of ice or glacier cover. |
| Greening | An increase in the amount of photosynthetically active pigments (primarily chlorophylls) in vegetation. |
| Increased sediment load | Increase in the amount of a substance discharged into a body of water (e.g., salt or sediment). |
| Inundation | Progressive submergence of land or structures by water. |
| Invasive or exotic species | A plant or animal that has been introduced into a region (terrestrial or aquatic) in which it does not naturally occur and that becomes established and spreads, displacing naturally occurring species. |
| Lava flow | A mass of molten rock produced during the eruption of a volcano and moving down its sides. |
| Leaf scorch | A non-infectious, physiological condition caused by unfavourable environmental situations. It is not caused by fungus, bacteria, or virus. The problem may appear on almost any plant if weather conditions are favourable, such as high temperatures, dry winds, salt carrying winds and low soil moisture. |
| Mine abandonment | Land formally used for extractive industry but no longer in use and no new use observed. |
| Mine construction | Creation of infrastructure in preparation for mining. |
| Mine expansion | Increase in the area of infrastructure associated with extraction of minerals or materials |
| Natural surface gain | Increase in the area occupied by a natural surface. |
| Natural surface loss | Decrease in area of natural surface. |
| Net snow gain (extent) | Progressive gain of snow extent. |
| Net snow gain (hydroperiod) | The increase in the residence time of snow cover, often expressed as a percentage of a year. |
| Net snow loss (extent) | Loss of snow cover through melt resulting in exposure of the underlying surface. |
| Net snow loss (hydroperiod) | The decrease in the residence time of snow cover, often expressed as a percentage of a year. |
| Phenological change | Alteration in the timing of natural lifecycle events. |
| Railway or road abandonment | Transport infrastructure no longer in use and no new use observed. |
| Railway or road construction | Creation of transport infrastructure. |
| Receding flood | Movement of water away from an area after a flood. |
| Salinisation | The process of soils or water becoming saltier. |
| Sea ice decrease | Decrease in the extent and/or volume of frozen marine water. |
| Sea ice increase | Increase in the extent and/or volume of frozen marine water. |
| Sea level fall | A decrease in the mean level of the oceans. Relative sea level decrease occurs where there is a local decrease in the level of the ocean relative to the land, which might be caused by ocean falling, the land rising, or both. |
| Sea level rise | An increase in the mean level of the oceans. Relative sea level increase occurs where there is a local increase in the level of the ocean relative to the land, which might be caused by ocean rising, the land subsiding, or both. In areas with rapid land level uplift (e.g., seismically active areas), relative sea level can fall. |
| Sedimentation | Deposition of sediment from flowing water (in channels or floodplains) or standing water (in wetlands, lakes, or oceans). |
| Sinkhole | A sinkhole is a closed natural depression in the ground surface caused by removal of material below the ground and either collapse or gradual subsidence of the surface into the resulting void. |
| Snow accumulation | Gradual gathering of snow over an area already covered in snow. |
| Snow melt | Loss of snow cover due to heat. |
| Urban damage | Damage to urban buildings and infrastructure. |
| Urban decay | The process of deterioration in the integrity of buildings and infrastructure. |
| Urban densification | Increases in the number of buildings and associated infrastructure as human populations move into an area to live or work. |
| Urban development | Economic, social and political changes that improve the wellbeing of people in an urban setting. |
| Urban growth | Increase in extent of urban land cover through the construction of buildings and associated infrastructure. |
| Urban loss (extent) | Decrease in area of artificial surfaces (buildings and infrastructure). |
| Urban renewal | Redevelopment of a built environment to address urban decay. |
| Urban sprawl | Unplanned low-density development surrounding an urban area that often starts as rural land. |
| Vegetation damage | Physical harm that impairs the value, usefulness, or normal function of plants or plant communities. |
| Vegetation dieback | Mortality of all or part of plant components, either singularly or en masse. |
| Vegetation gain (amount) | Expansion of whole plant communities into an area. This includes native vegetation. |
| Vegetation gain (extent) | An increase in the area occupied by plants. |
| Vegetation health deterioration | Deterioration in the state or function of plants or plant communities. |
| Vegetation health improvement | Improvement in the state or function of plants or plant communities. |
| Vegetation loss (extent) | Loss of vegetation communities from an area. |
| Vegetation reduction (amount) | Reduction in the diversity, abundance and/or coverage of plants within an area. |
| Vegetation reduction in understory (amount) | Reduction in the diversity, abundance and/or coverage of subcanopy plants within an area. |
| Vegetation species change | Changes in the floristic composition of vegetation in an area. |
| Water depth decrease | Decrease in the amount of water from an existing water body. |
| Water depth increase | Increase in the amount of water within an existing water body. |
| Water gain (extent) | Increase in the extent of water from the bounds of an existing water body. |
| Water loss (extent) | Decrease in the extent of water from the bounds of an existing water body. |
| Water movement change | Change in liquid water currents or turbulence. |
| Water quality change | Change in the physical, chemical and biological characteristics of water and the measure of its condition relative to the requirements for one or more biotic species and/or to any human need or purpose. |

Table 3: Web of science records (71949; 40158 cross-linked between categories) associated with the term land cover change (from 1945-2021).

| Web of Science Categories | records | | % of 40158 | |  |
| --- | --- | --- | --- | --- | --- |
| Environmental sciences | 13945 | | 34.7 | |  |
| Geosciences multidisciplinary | 7828 | | 19.5 | |  |
| Remote sensing | 6841 | | 17 | |  |
| Ecology | 6569 | | 16.4 | |  |
| Imaging science photographic technology | 4274 | | 10.6 | |  |
| Geographical physical | 3902 | | 9.7 | |  |
| Water resources | 3815 | | 9.5 | |  |
| Meteorology/atmospheric sciences | 3603 | | 9 | |  |
| Environmental studies | 3132 | | 7.8 | |  |
| Biodiversity conservation | 2329 | | 5.8 | |  |
| Geography | 1894 | | 4.7 | |  |
| Forestry | 1744 | | 4.3 | |  |
| Soil science | 1481 | | 3.7 | |  |
| Multidisciplinary sciences | 1406 | | 3.5 | |  |
| Engineering electrical electronic | 1265 | | 3.2 | |  |
| Green sustainable science technology | 1108 | | 2.8 | |  |
| Engineering environmental | 1089 | | 2.7 | |  |
| Plant sciences | 856 | | 2.1 | |  |
| Agricultural multidisciplinary | 847 | | 2.1 | |  |
| Engineering civil | 837 | | 2.1 | |  |
| Urban studies | 723 | | 1.8 | |  |
| Agronomy | 691 | | 1.7 | |  |
| Regional urban planning | 672 | | 1.7 | |  |
| Geochemistry geophysics | 555 | | 1.4 | |  |
| Marine freshwater biology | 543 | | 1.4 | |  |
|  | |  | |  | |

Table 4. Summary of main terms relating to land cover classifications and descriptions.

| Term | Definition and associated information |
| --- | --- |
| Environment | The surroundings in which an organism lives, including the air, water, food and energy required for its survival. |
| Ecosystem | A community consisting of both organisms and non-living components (e.g., weather, landscape) working together to create a successful living space. |
| Overarching Environmental Descriptors | Land cover classes that provide the initial broad divisions of the dominant cover (e.g., croplands, urban, water). |
| Essential Environmental Descriptors | Categorical characteristics that are essential for delivering land cover classifications according to pre-defined taxonomies. |
| Additional Environmental Descriptors | Categorical or continuous characteristics that are external to a land cover taxonomy, playing no part in its construction but augmenting information. |
| Abiotic drivers | Associated primarily with climate and weather and soil/geomorphological factors (e.g., nutrient supply). |
| Biotic drivers | Relate to changes in the distribution, abundance and/or types of flora and fauna |
| Human influenced drivers | Anthropogenic in origin and including include economic activity, changes in population or the need to conserve, protect or restore the environment |
| Extent | The area of a land cover class (e.g., ha, km^2^) |
| Amount | The physical, chemical or biological quantity of material (e.g., canopy cover (%) or water depth (m)) |
| Amount (intensive property) | A quantity where the value does not depend on the amount of substance nor the extent of a system (e.g., water turbidity, NTU, salinity, g kg^-1^). |
| Amount (extensive property) | A quantity that is proportional to the size of the system (e.g., above ground biomass (Mg ha^-1^), photosynthetic vegetation fraction (%) and water depth (m). |
| Type | A class assigned to a physical material (e.g., species, broad land cover). |
| Environmental condition | The quality of components of the environment measured in terms of its physical, chemical and biological characteristics. |
| Ecosystem condition | The quality or health of an ecosystem measured in terms of its physical, chemical and biological characteristics of ecological systems and the processes and interactions that connect them. |

Table 5. Summary of main terms relating to temporal changes associated with natural events or processes or human activities.

| Term | Definition and associated information |
| --- | --- |
| Occurrence | The time span of the actual natural event or process or human activity |
| Lag | The time between commencement and detection |
| Manifestation | The time period of detectability |
| Duration | The time from commencement to completion of a natural event or process or human activity. |
| Required evidence | Evidence that is needed to make the decision on the allocation of a change category |
| Confirmatory evidence | Evidence that reinforces a decision |
| Time dependence | Where the impacts (e.g., snow accumulation) and/or pressures (e.g., flash floods, crop rotation) have some link to measures of time. |
| Time independence | Impacts (e.g., dieback) and/or pressures (e.g., ecological restoration) not directly linked to a time measurement. |
| Simultaneous change | Concurrent losses, gains or transitions in land cover |
| Sequential change | Losses, gains or transitions in land cover that follow on from each other. |
